# BiFC and FACS-based CRISPR screening revealed that QKI promotes PABPN1 LLPS in colorectal cancer cells

**DOI:** 10.1093/procel/pwaf022

**Published:** 2025-03-06

**Authors:** Mengxia Li, Zhijie Hu, Yingye Huang, Yuting Han, Cheng Liang, Yuchi Liu, Runze Wu, Xin Lu, Ke Deng, Susu Liu, Xin Ou, Yuwei Li, Chao Liu, Xuening Li, Jingting Liang, Yonggui Fu, Anlong Xu

**Affiliations:** State Key Laboratory of Biocontrol, Guangdong Province Key Laboratory of Pharmaceutical Functional Genes, Department of Biochemistry, Innovation Center for Evolutionary Synthetic Biology, School of Life Sciences, Sun Yat-sen University, Guangzhou 510275, China; Department of Hepatology, Guangzhou Institute of Clinical Medicine of Infectious Diseases, Guangzhou Eighth People’s Hospital, Guangzhou Medical University, Guangzhou 510440, China; State Key Laboratory of Biocontrol, Guangdong Province Key Laboratory of Pharmaceutical Functional Genes, Department of Biochemistry, Innovation Center for Evolutionary Synthetic Biology, School of Life Sciences, Sun Yat-sen University, Guangzhou 510275, China; State Key Laboratory of Biocontrol, Guangdong Province Key Laboratory of Pharmaceutical Functional Genes, Department of Biochemistry, Innovation Center for Evolutionary Synthetic Biology, School of Life Sciences, Sun Yat-sen University, Guangzhou 510275, China; State Key Laboratory of Biocontrol, Guangdong Province Key Laboratory of Pharmaceutical Functional Genes, Department of Biochemistry, Innovation Center for Evolutionary Synthetic Biology, School of Life Sciences, Sun Yat-sen University, Guangzhou 510275, China; State Key Laboratory of Biocontrol, Guangdong Province Key Laboratory of Pharmaceutical Functional Genes, Department of Biochemistry, Innovation Center for Evolutionary Synthetic Biology, School of Life Sciences, Sun Yat-sen University, Guangzhou 510275, China; State Key Laboratory of Biocontrol, Guangdong Province Key Laboratory of Pharmaceutical Functional Genes, Department of Biochemistry, Innovation Center for Evolutionary Synthetic Biology, School of Life Sciences, Sun Yat-sen University, Guangzhou 510275, China; State Key Laboratory of Biocontrol, Guangdong Province Key Laboratory of Pharmaceutical Functional Genes, Department of Biochemistry, Innovation Center for Evolutionary Synthetic Biology, School of Life Sciences, Sun Yat-sen University, Guangzhou 510275, China; State Key Laboratory of Biocontrol, Guangdong Province Key Laboratory of Pharmaceutical Functional Genes, Department of Biochemistry, Innovation Center for Evolutionary Synthetic Biology, School of Life Sciences, Sun Yat-sen University, Guangzhou 510275, China; State Key Laboratory of Biocontrol, Guangdong Province Key Laboratory of Pharmaceutical Functional Genes, Department of Biochemistry, Innovation Center for Evolutionary Synthetic Biology, School of Life Sciences, Sun Yat-sen University, Guangzhou 510275, China; State Key Laboratory of Biocontrol, Guangdong Province Key Laboratory of Pharmaceutical Functional Genes, Department of Biochemistry, Innovation Center for Evolutionary Synthetic Biology, School of Life Sciences, Sun Yat-sen University, Guangzhou 510275, China; State Key Laboratory of Biocontrol, Guangdong Province Key Laboratory of Pharmaceutical Functional Genes, Department of Biochemistry, Innovation Center for Evolutionary Synthetic Biology, School of Life Sciences, Sun Yat-sen University, Guangzhou 510275, China; State Key Laboratory of Biocontrol, Guangdong Province Key Laboratory of Pharmaceutical Functional Genes, Department of Biochemistry, Innovation Center for Evolutionary Synthetic Biology, School of Life Sciences, Sun Yat-sen University, Guangzhou 510275, China; State Key Laboratory of Biocontrol, Guangdong Province Key Laboratory of Pharmaceutical Functional Genes, Department of Biochemistry, Innovation Center for Evolutionary Synthetic Biology, School of Life Sciences, Sun Yat-sen University, Guangzhou 510275, China; State Key Laboratory of Biocontrol, Guangdong Province Key Laboratory of Pharmaceutical Functional Genes, Department of Biochemistry, Innovation Center for Evolutionary Synthetic Biology, School of Life Sciences, Sun Yat-sen University, Guangzhou 510275, China; State Key Laboratory of Biocontrol, Guangdong Province Key Laboratory of Pharmaceutical Functional Genes, Department of Biochemistry, Innovation Center for Evolutionary Synthetic Biology, School of Life Sciences, Sun Yat-sen University, Guangzhou 510275, China; State Key Laboratory of Biocontrol, Guangdong Province Key Laboratory of Pharmaceutical Functional Genes, Department of Biochemistry, Innovation Center for Evolutionary Synthetic Biology, School of Life Sciences, Sun Yat-sen University, Guangzhou 510275, China; Sun Yat-sen University Institute of Advanced Studies, Hong KongSAR, China

**Keywords:** liquid–liquid phase separation, PABPN1, CRISPR/Cas9 screening, colorectal cancer, QKI

## Abstract

Protein liquid–liquid phase separation (LLPS), a pivotal phenomenon intricately linked to cellular processes, is regulated by various other proteins. However, there is still a lack of high-throughput methods for screening protein regulators of LLPS in target proteins. Here, we developed a CRISPR/Cas9-based screening method to identify protein phase separation regulators by integrating bimolecular fluorescence complementation (BiFC) and fluorescence-activated cell sorting (FACS). Using this newly developed method, we screened the RNA-binding proteins that regulate PABPN1 phase separation and identified the tumor suppressor QKI as a promoter of PABPN1 phase separation. Furthermore, QKI exhibits decreased expression levels and diminished nuclear localization in colorectal cancer cells, resulting in reduced PABPN1 phase separation, which, in turn, promotes alternative polyadenylation (APA), cell proliferation, and migration in colorectal cancer.

## Introduction

The assembly of various proteins and RNAs into condensates driven by weak multivalent interactions is known as liquid–liquid phase separation (LLPS). LLPS plays an important role in diverse membraneless structures and participates in numerous biological processes (Banani et al., 2017; Wang et al., 2021). Abnormal phase separation of several proteins has also been reported to be implicated in diseases (Wang et al., 2021). For example, the aberrant aggregation of proteins such as FUS, Tau, and TDP-43 has been linked to the pathogenesis of neurodegenerative disorders (Zbinden et al., 2020). Similarly, mutant SHP2 has been shown to recruit and activate wild-type SHP2 within aggregates, thereby facilitating MAPK activation and promoting tumorigenesis (Zhu et al., 2020). Extensive studies have demonstrated that other proteins can regulate the LLPS of target proteins, thereby influencing cellular activities (Hur et al., 2020; Zhang et al., 2018). Screening and validating new regulators are crucial for understanding the mechanisms underlying LLPS-mediated cellular activities and for developing novel therapeutic approaches. There is an urgent need for a high-throughput method to identify new LLPS regulators.

Currently, large-scale screening of small-molecule drugs or sgRNA libraries for the regulation of LLPS is primarily conducted using high-content imaging. For instance, ET516 was identified as a compound that specifically disrupts androgen receptor condensates through a phase-separation-based phenotypic screen (Xie et al., 2022). DropScan, a high-throughput screening method, was established and used to identify small molecules capable of dissolving condensates formed by FET–ETS oncofusion proteins (Wang et al., 2023). Similarly, Tau-BiFC combined with high-content image analysis revealed levosimendan as a new anti-tau agent (Lim et al., 2023). Regarding sgRNA libraries, the CRaft-ID method was developed to screen for regulators affecting stress granules by isolating microrafts containing genetic clones harboring individual guide RNAs, followed by high-content image analysis (Wheeler et al., 2020). However, the limitations of high-content imaging techniques—including challenges in sample preparation, high screening costs, and complex data processing, particularly for sgRNA library screening—restrict their widespread application.

PABPN1, a 3′ end processing factor, undergoes phase separation and plays an important role in alternative polyadenylation (APA). PABPN1 phase separation exists in several states, including no LLPS, liquid droplets, and fibril-like aggregates, making it a suitable candidate for testing newly developed methods. PABPN1-mRNA condensates lead to the assembly of NPADs (nuclear poly(A) domains) in mouse growing oocytes, promoting RNA processing and the generation of long 3′ UTR transcripts, which was crucial for mouse oocyte development and female reproduction (Dai et al., 2022). Additionally, poly(A) RNA has been shown to promote the aggregation of PABPN1 condensates (Dai et al., 2022). We previously found that the LLPS droplets of PABPN1 were significantly reduced in colorectal cancer, which promoted the usage of proximal poly(A) sites of CTNNBIP1 and increased cell proliferation and migration (Hu et al., 2024). The splicing factor SNRPD2, which is upregulated in colorectal cancer, disrupts PABPN1 LLPS by interacting with PABPN1 (Hu et al., 2024). Additionally, another U1 snRNP core protein, SNRNP70, was found to promote PABPN1 LLPS to form aggregates, thereby attenuating its inhibitory effect on proximal poly(A) sites (Hu et al., 2022). Furthermore, alanine-expanded mutant PABPN1 leads to the formation of toxic aggregates, resulting in oculopharyngeal muscular dystrophy (OPMD) (Banerjee et al., 2013). Collectively, these findings suggest that PABPN1 is involved in various physiological and pathological processes through alterations in its LLPS. Thus, identifying regulators of PABPN1 LLPS is essential for further understanding its functional and regulatory mechanisms.

Here, we developed a high-throughput platform for screening regulators of phase separation by integrating CRISPR/Cas9, bimolecular fluorescence complementation (BiFC), fluorescence-activated cell sorting (FACS), and second-generation sequencing techniques. Using this new approach, we screened for regulators of PABPN1 LLPS and identified that QKI can promote its LLPS. Decreased expression levels and reduced nuclear localization of QKI in colorectal cancer cells attenuate PABPN1 LLPS, thereby promoting proximal APA sites, cell proliferation, and migration.

## Results

### Fluorescence intensity of BiFC can quantify the LLPS changes of PABPN1

**Figure 1.**
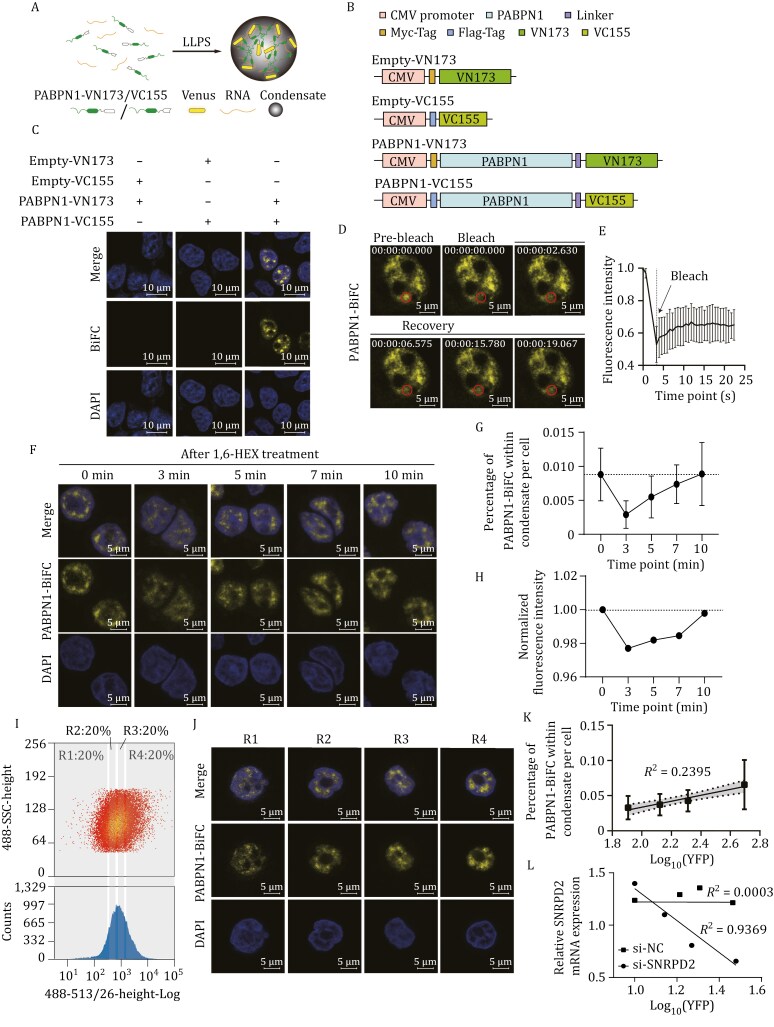
**Fluorescence intensity of PABPN1-BiFC reflects the changes of PABPN1 LLPS.** (A) Schematic representation of Venus reconstruction driven by PABPN1 LLPS. (B) Schematic diagram of PABPN1-BiFC constructs. (C) Representative immunofluorescence (IF) images show the combination of PABPN1-VN173/PABPN1-VC155 can produce BiFC signals in HEK293T cells. HEK293T cells were transfected with Empty-VN173/Empty-VC155 or PABPN1-VN173/PABPN1-VC155 constructs. Nuclei were stained with DAPI. (D) IF images illustrate the recovery of fluorescence after photobleaching of PABPN1-BiFC. HEK293T cells were co-transfected with Myc-PABPN1-VN173 and Flag-PABPN1-VC155. The outlined circle indicates changes in the droplets. (E) The curve represents the recovery of normalized fluorescence intensity. Data are presented as mean ± SD. *n* = 8. (F) The representative confocal microscopy images of SW480-PABPN1-BiFC upon treatment with 1.5% 1,6-HEX at different time points (0, 3, 5, 7, 10 min). (G) The percentage of PABPN1-BiFC within condensate after treatment with 1.5% 1,6-HEX at different time points. *n* = 17. (H) Fluorescence intensity of PABPN1-BiFC measured by flow cytometry analysis at different time points under 1,6-HEX treatment. (I) The representative FACS profiles of cells with stably expressed SW480-PABPN1-BiFC. The cells were fractioned into four groups (R1, R2, R3, and R4). (J) Confocal microscopy images of SW480 cells from four groups of panel (I). (K) The positive correlation between the fluorescence intensity of PABPN1-BiFC and the percentage of PABPN1-BiFC within condensate of the cells from four groups in panel (I). The data are presented as mean ± SD. *n* = 20. *P <* 0.0001. 95% CI of the slop: 0.02530–0.05927. (L) The negative correlation between expression level of SNRPD2 and fluorescence intensity of PABPN1-BiFC. SW480 cells stably expressed PABPN1-BiFC were transfected with si-SNRPD2, and sorted with FACS as panel (I). Relative expression levels of SNRPD2 were measured with qRT-PCR and si-NC was used as negative control.

To identify factors regulating protein LLPS, we applied an indicator based on BiFC of Venus fluorescent protein. The two split fragments of VN173 (1–173 aa) and VC155 (156–228 aa) of Venus protein have been developed for BiFC assays (Shyu et al., 2006). In principle, VN173 and VC155 fragments should reassemble and emit fluorescence when they are brought into proximity within condensates (Fig. 1A). We fused the two fragments, respectively, to either the N-terminus or C-terminus of PABPN1 and transfected them into HEK293T cells (Fig. S1A). We found that only fusing both fragments to the C-terminus of PABPN1 does not interfere with the formation of droplet-like condensates of PABPN1 (Fig. S1B). Next, we co-transfected Empty-VN173/Empty-VC155 with PABPN1-VN173/ PABPN1-VC155 into HEK293T cells and observed fluorescence emission only in cells with co-transfection of PABPN1-VN173 and PABPN1-VC155 but not in other groups (Fig. 1B and 1C).

We then investigated the LLPS properties of the fusion protein condensates. FRAP assay shows that the fluorescence intensity of PABPN1-BiFC condensates partially recovers within 30 s (Fig. 1D and 1E). Confocal microscopy analysis reveals that PABPN1-BiFC condensates dissolves within 1–3 min and recovers within 3–10 min under 1,6-HEX treatment (Fig. 1F and 1G), consistent with the changes observed in endogenous PABPN1 LLPS (Fig. S2A and S2B). This matches the characteristic changes of LLPS droplets under short- and long-term 1,6-HEX treatment (Liu et al., 2021). Furthermore, flow cytometry analysis also shows a similar pattern for PABPN1-BiFC fluorescence intensity under 1,6-HEX treatment (Fig. 1H). These results demonstrate that PABPN1-VN173 and PABPN1-VC155 fusion proteins can undergo LLPS, and BiFC has the potential to quantify LLPS.

Next, we sought to determine whether BiFC fluorescence intensity could quantitatively evaluate the extent of phase separation of proteins. We established SW480 cell lines stably expressing Cas9 and PABPN1-BiFC (Fig. S3A and S3B). Using FACS, we sorted the stably expressed SW480 cells into four cell groups (R1, R2, R3, and R4) based on BiFC fluorescence intensity, with each group representing 20% of the total cell population (Fig. 1I). With confocal microscopy and ImageJ analysis, we measured the percentage of PABPN1-BiFC within condensates per cell for each group. A statistically significant linear relationship was observed between the mean fluorescence intensity of the four cell groups and the percentage of PABPN1-BiFC within condensates in each group (*R*² = 0.2395 and *P *< 0.0001) (Fig. 1J and 1K), indicating that the overall fluorescence intensity can serve as a metric for assessing changes in phase separation.

We previously found that SNRPD2 represses PABPN1 LLPS (Hu et al., 2024). Here, we used SNRPD2 as a positive control to validate the ability of BiFC and FACS in screening regulators of protein LLPS. We knocked down SNRPD2 expression using siRNA in the SW480 cell with stable expression of PABPN1-BiFC (Fig. S4A), and confocal microscopy analysis shows higher percentage of PABPN1-BiFC within condensate in cells with SNRPD2 knockdown than in control cells (*P < *0.01) (Fig. S4B and S4C), which confirms that SNRPD2 can repress the LLPS of PABPN1. Given the heterogeneity in knockdown efficiency among cells, we exploited this variability to analyze the association between SNRPD2 expression and PABPN1-BiFC fluorescence intensity. Cells with siRNA treatment were sorted into four groups based on PABPN1-BiFC fluorescence intensity using FACS, and the mean fluorescence intensity of PABPN1-BiFC was calculated for each group. SNRPD2 and PABPN1 expression levels in each group were measured using qRT-PCR. A significant correlation was observed between PABPN1-BiFC fluorescence intensity and SNRPD2 expression level in SNRPD2-knockdown samples (*R*^2^ = 0.9369), but not in the control samples treated with scramble siRNA (*R*^2^ = 0.0003) (Fig. 1L). Additionally, the correlation coefficient R^2^ between PABPN1 expression level and the PABPN1-BiFC fluorescence intensity was only 0.8573 (Fig. S4D), and 0.6532 between PABPN1 and SNRPD2 expression levels (Fig. S4E), indicating that SNRPD2 affects PABPN1-BiFC fluorescence intensity mainly by modulating PABPN1 LLPS. These results demonstrate that BiFC and FACS analyses can be effectively used to screen new regulators of PABPN1 LLPS.

### Screening new regulators of PABPN1 LLPS

**Figure 2.**
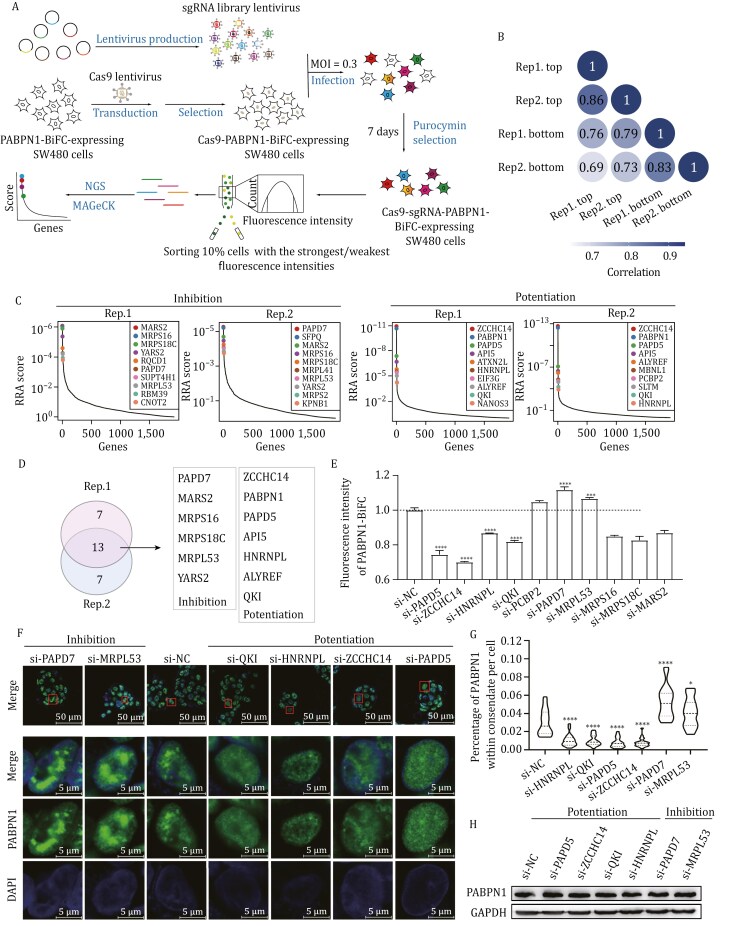
**CRISPR screening and validation of PABPN1 phase separation regulators.** (A) Schematic diagram of the workflow for screening the regulators of PABPN1 LLPS. (B) Pairwise Pearson correlation *r* of the normalized read numbers of each sgRNA among the samples. (C) Ranked RRA scores for each gene calculated by MAGeCK. Left: the inhibition protein. Right: the potentiation proteins. (D) Venn diagram of top 10 candidate inhibition and potentiation genes in two replicates. (E) The fluorescence intensities of PABPN1-BiFC in cells with knockdown of candidate genes measured by flow cytometry analysis. Candidate genes were knocked down using siRNA in SW480 cells, and si-NC was used as a control. Three biological replicates were performed. Data are presented as mean ± SD. ***P < 0.001 and ****P < 0.0001 with *t*-test. (F) IF images illustrate endogenous PABPN1 LLPS in cells with knockdown of candidate genes. SW480 were transfected with siRNA targeting candidate genes and then stained with anti-PABPN1 antibodies and DAPI. (G) The percentage of PABPN1 within the condensate in cells with knocked-down candidate genes in panel (F). *n* = 20. *P < 0.05 and ****P < 0.0001 with *t*-test. (H) The expression level of PABPN1 protein in cells after interfering with the candidate gene detected by Western blot. SW480 cells were transfected with siRNA targeting candidate genes.

Next, we integrated CRISPR/Cas9, BiFC, FACS, and second-generation sequencing to screen regulators of PABPN1 LLPS using SW480 cells stably expressing Cas9 and PABPN1-BiFC (Fig. 2A). A lentiviral sgRNA library was constructed, comprising 8,665 sgRNAs targeting 1,484 human RBP genes (with 3–6 sgRNAs per gene), along with 434 control sgRNAs (Table S1). The control sgRNAs, which constitute approximately 5% of the library, include randomly designed scramble sequences that do not target human genes. SW480 cells were transfected with the lentiviral sgRNA library at a multiplicity of infection (MOI) of 0.3 and were selected via puromycin treatment. Subsequently, using FACS, we collected the top and bottom 10% fractions of cells based on fluorescence intensity. The DNA fragments encoding sgRNAs were then amplified with a pair of primers containing barcodes (Fig. S5), followed by sequencing with Illumina and analysis using MAGeCK. Two biological replicates were performed.

We obtained about 1 × 10^7^ reads for each sample. We first normalized the read numbers of each sgRNA using the median ratio method, and calculated pairwise correlation coefficients among samples based on these values. The results show higher correlation between replicates than between cell fractions (Figs. 2B and S6A), indicating good reproducibility of the experiments. We then calculated the fold change of each sgRNA between the top and bottom fractions and obtained the statistically significant values using a learned mean-variance model. There are 807 and 617 sgRNAs with significant changes (FDR < 0.01) between the two fractions for the two replicates, respectively, and the union contains 1,248 sgRNAs. We also calculated the correlation of the fold change of sgRNAs between two replicates, and the results show that union significant sgRNAs have higher correlation (*R* = 0.19) between replicates than other sgRNAs (*R* = 0.049) (*P *= 0 with a permutation test) (Fig. S6B).

Robust rank aggregation (RRA) scores based on the FDR of sgRNAs targeting each gene were calculated (Fig. 2C) (Li et al., 2014). Among the genes with the top 10 RRA scores in the inhibition and potentiation lists from each replicate, there are 13 overlapped genes (Fig. 2D). For SNRPD2, four of six sgRNAs show increased read counts in the Top fraction compared to the Bottom, although it is not included in the top gene list (Fig. S7).

We subsequently selected five candidate genes associated with potentiation and five with inhibition to validate their effects on PABPN1 LLPS through siRNA-mediated knockdown (Fig. S8). The flow cytometry result reveals that knockdown of PAPD5, ZCCHC14, HNRNPL and QKI can reduce the fluorescence intensity of PABPN1-BiFC by about 13%–30% (*P < *0.0001), while knockdown of PAPD7 and MRPL53 can increase it by about 6%–11% (*P <* 0.0001 or *P < *0.001) (Fig. 2E). Immunofluorescence (IF) assay with anti-PABPN1 confirms that knockdown of PAPD7 and MRPL53 can significantly promote PABPN1 LLPS (*P <* 0.0001 or *P <* 0.05), while knockdown of PAPD5, ZCCHC14, HNRNPL and QKI can inhibit it (*P <* 0.0001) (Fig. 2F and 2G). Importantly, knockdown of these candidate genes does not affect the expression level of endogenous PABPN1 (Fig. 2H).

### The tumor suppressor QKI-6 promotes PABPN1 LLPS

**Figure 3.**
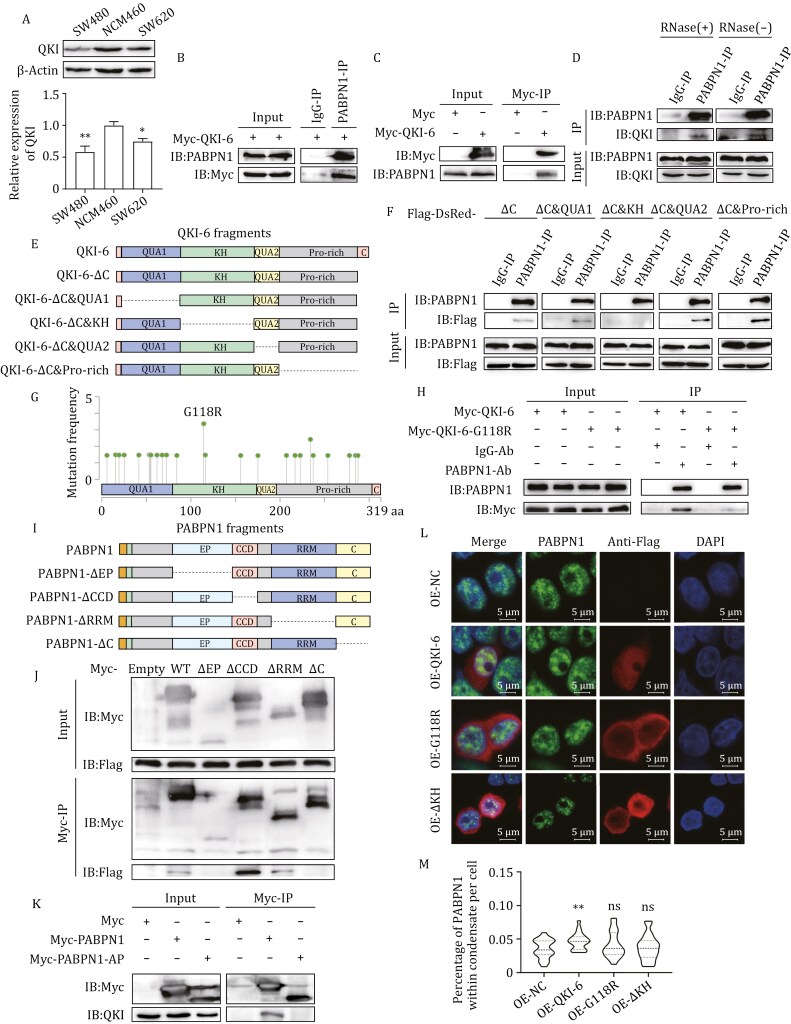
**The tumor suppressor QKI promotes PABPN1 LLPS through its KH domain.** (A) Lower protein expression level of QKI in colorectal tumor cell lines SW480 and SW620 than normal cell line NCM460. Top: representative Western blot image showing endogenous QKI expression in SW480, NCM460, and SW620 cells. Bottom: quantitative analysis of band grayscale intensity. *n* = 3. *P < 0.05 and **P < 0.01 with *t*-test. (B and C) Co-IP with anit-PABPN1 (B) and anti-Myc (C) reveals the interaction between Myc-QKI-6 and endogenous PABPN1. HEK293T cells were transfected with Myc-QKI-6. (D) Co-IP analysis reveals the interaction between endogenous QKI and PABPN1 with and without RNase treatment. (E) A schematic representation of the domain deletion mutants of QKI: QUA1 (1–81 aa), KH (82–181 aa), QUA2 (182–205 aa), Pro-rich (206–311 aa), and C-Terminal (312–319 aa). (F) Co-IP with anti-PABPN1 shows that KH domain of QKI-6 is responsible for the interaction between QKI-6 and PABPN1. HEK293T cells were transfected with Flag-DsRed-tagged QKI fragments. (G) The recurrent mutation of G118R in KH domain of QKI-6 in colorectal cancer patients from cBioPortal databases. (H) Co-IP assay reveals that the G118R mutation of QKI-6 can disrupt the interaction between QKI-6 and PABPN1. (I) A schematic representation of the domain deletion mutants of PABPN1: Ala (1–11 aa), EP (51–118 aa), CCD (119–146 aa), RRM (162–294 aa) and C-Terminal (295–306 aa). (J) Co-IP reveals that EP and C-terminal domains of PABPN1 are responsible for the interaction between PABPN1 and QKI-6. Myc-tagged PABPN1 deletion mutants and Flag-QKI-6 were transfected into HEK293T cells. (K) Glutamate to alanine mutations in the EP domain attenuates the interaction between PABPN1 and QKI. (L) Representative IF images illustrating the effect of wild-type or mutant of QKI-6 on PABPN1 LLPS in SW480 cells. Cells were transfected with CMV-Flag, Flag-QKI-6, Flag-QKI-6-ΔKH, or Flag-QKI-6-G118R. After 24 h, the cells were stained using anti-Flag and anti-PABPN1 antibodies. (M) Quantification of the percentage of PABPN1 within condensate in SW480 cells of panel (L). *n* = 20. ns, no significance and **P < 0.01 with *t*-test.

Among the candidate genes, QKI has been reported to be a colorectal cancer tumor suppressor (Yang et al., 2010). TCGA data also show that QKI expression is significantly downregulated in several types of cancer tissues including colorectal cancer (Fig. S9A and S9B). Western blot further confirms a substantial decrease in QKI protein levels in colorectal cancer cell lines (SW480 and SW620) compared with the normal cell line (NCM460) (Fig. 3A).

To investigate the mechanism by which QKI regulates PABPN1 LLPS, we first explored the interaction between QKI and PABPN1. The QKI gene produces three major isoforms of mRNA (QKI-5, QKI-6, and QKI-7) through APA and alternative splicing, with the protein isoforms differing only in their C-terminal regions. We overexpressed the three QKI isoforms tagged with Myc in HEK293T cells, and Co-IP assay with anti-Myc antibody shows that all QKI isoforms can pull down PABPN1 (Fig. S10A and S10B). Given the essential role of QKI-6 in colorectal cancer (Yang et al., 2010), we selected QKI-6 for further study. Co-IP assay with HEK293T cells overexpressing Myc-QKI-6 confirms that Myc-QKI-6 and PABPN1 can pull down each other using both anti-Myc and anti-PABPN1 antibodies (Fig. 3B and 3C). Moreover, PABPN1 can also pull down endogenous QKI in the presence or absence of RNase treatment (Fig. 3D), suggesting that RNA is not required for the interaction between PABPN1 and QKI.

To identify the critical domain of QKI-6 for this interaction, we generated five domain deletion mutants of QKI-6 fused with Flag-DsRed at their N-terminus (Zhao et al., 2023) and transfected them into HEK293T cells (Fig. 3E). Since all three QKI isoforms can pull down PABPN1, we deleted the C-terminal region in all five mutants. Co-IP assay with anti-PABPN1 shows that only QKI-6-ΔKH fails to interact with PABPN1, highlighting the critical role of the KH domain in this interaction (Fig. 3F). Using the cBioPortal database, we found a recurrent mutation (G118R) in the KH domain of QKI-6 in colorectal cancer patients (Fig. 3G). Co-IP analysis reveals that the Myc-QKI-6-G118R almost completely loses the ability to interact with PABPN1 (Fig. 3H).

We next constructed four domain deletion mutants of PABPN1 to identify the regions required for its interaction with QKI-6 (Fig. 3I), and co-transfected the Myc-tagged PABPN1 deletion mutants and Flag-tagged QKI-6 into HEK293T cells. Co-IP assay with anti-Myc shows that the ΔEP and ΔC-terminal mutants of PABPN1 lose the ability to interact with QKI-6 (Fig. 3J). Our previous work demonstrated that the EP domain of PABPN1 plays a key role in LLPS by interacting with SNRPN70 and SNRPD2 (Hu et al., 2022, 2024). Here, we then mutated all glutamine residues in the EP domain to alanines, generating the PABPN1-AP mutant (Fig. S11). Overexpression of Myc-tagged PABPN1-WT and PABPN1-AP in HEK293T cells followed by Co-IP analysis with anti-Myc reveals that PABPN1-AP fails to pull down endogenous QKI (Fig. 3K). This indicates that mutation of the EP domain disrupts the interaction between QKI and PABPN1. These findings suggest that the KH domain of QKI and the EP and C-terminal domains of PABPN1 mediate the interaction between PABPN1 and QKI.

We then transfected Flag-tagged QKI-6-WT, QKI-6-G118R, and QKI-6-ΔKH into SW480 and SW620 cells, respectively, to investigate the effects of mutation on PABPN1 LLPS. IF assay using anti-Flag and anti-PABPN1 antibody reveals that both mutants lose the ability to promote PABPN1 LLPS (Figs. 3L and S12A), while QKI-6-WT can significantly increase the percentage of PABPN1 within condensate (*P < *0.01) (Figs. 3M and S12B). These findings indicate that the interaction between QKI and PABPN1 can promote PABPN1 LLPS.

Next, we explored how the interaction can promote PABPN1 LLPS. RNA is usually a critical factor for LLPS of RBPs (Fay and Anderson, 2018). We expressed and purified the recombinant protein EGFP-PABPN1 and mixed it at a final concentration of 5 μmol/L with 10% PEG8000 and varying concentrations of total RNA (0, 100, and 200 ng/μL). Confocal imaging reveals that higher RNA concentration leads to larger and more droplets of PABPN1 (Fig. S13A). These results indicate that RNA can also promote the phase separation of PABPN1.

We identified the genes with binding peaks of PABPN1 from eCLIP data (Van Nostrand et al., 2020), and chose three genes of CTNNBIP1, E2F1 and CCND1 (Fig. S13B) to assess the effect of QKI on the RNA binding ability of PABPN1. We co-transfected SW480 cells with either CMV-Flag or Flag-QKI-6, along with Myc-PABPN1, and subsequently performed RNA immunoprecipitation with anti-Myc. qRT-PCR using primers targeting 3′ UTR of the three target genes show that overexpression of Flag-QKI-6 can significantly increase the enrichment of the mRNAs compared to CMV-Flag overexpression (Fig. S13C). These suggest that QKI-6 enhances the RNA-binding affinity of PABPN1, which may promote PABPN1 LLPS.

### QKI-6 regulates APA and cell proliferation and migration through PABPN1

**Figure 4.**
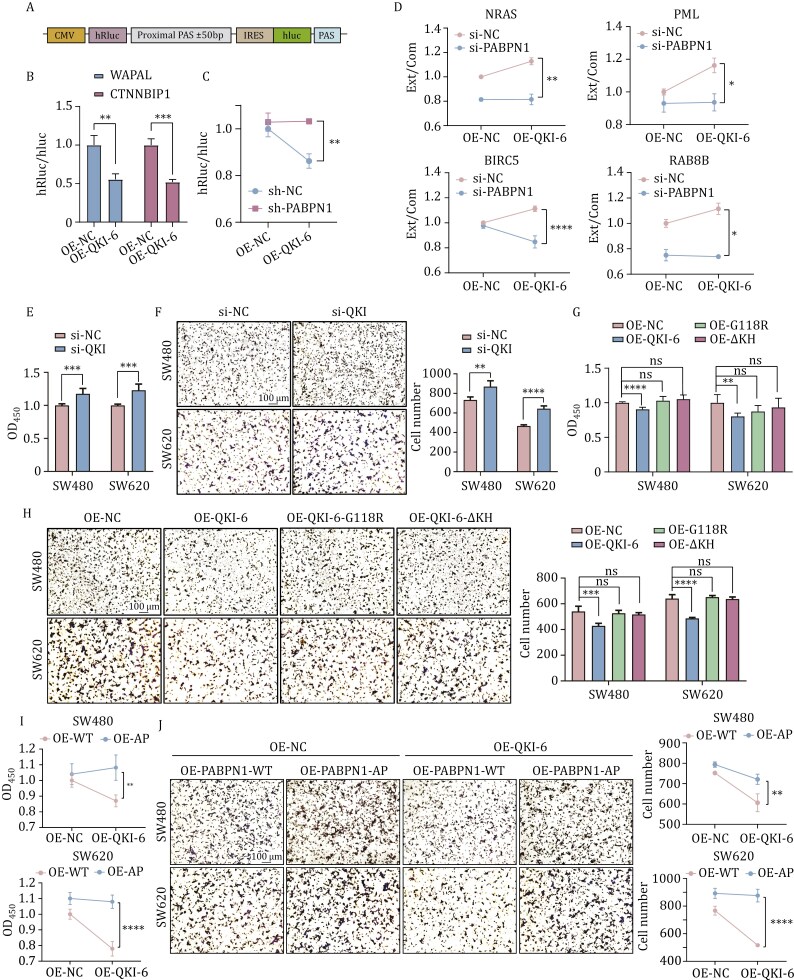
**QKI-6 regulates APA and cell proliferation and migration through PABPN1.** (A) Structural diagram of the dual luciferase reporter system. The WAPAL/CTNNBIP1 proximal poly(A) sites ± 50 bp were incorporated into the reporter system. (B) QKI-6 inhibits the usage of proximal poly(A) sites of PABPN1 target genes measured by dual-luciferase reporter assay. HEK293T cells were co-transfected with pCMV-Myc or Myc-QKI-6 and bicistronic-WAPAL/CTNNBIP1-PAS ± 50 bp constructs. *n* = 4. **P < 0.01 and ***P < 0.001 with *t*-test. (C) A significant interaction effect between QKI-6 and PABPN1 on CTNNBIP1 APA regulation, as measured by the dual luciferase reporter system. *n* = 4. **P < 0.01 with two-way ANOVA test. (D) QKI-6 inhibits the usage of proximal poly(A) site of endogenous genes through interaction with PABPN1. HEK293T cells were transfected with CMV-Flag or Flag-QKI-6, along with si-NC or si-PABPN1. After 48 h, qRT-PCR was performed to measure the expression level of extended and common region of 3′ UTRs of NRAS, PML, BIRC5, and RAB8B. *n* = 3. *P < 0.05, **P < 0.01 and ****P < 0.0001 with two-way ANOVA test. (E and F) CCK-8 (E, *n* = 6) and Transwell migration assays (F, *n* = 5) show the effects of QKI knockdown on the proliferation and migration of SW480 and SW620 cells. **P < 0.01, ***P < 0.001 and ****P < 0.0001 with *t*-test. (G and H) CCK-8 (G, *n* = 6) and Transwell migration assays (H, *n* = 5) show that deletion or mutation of the KH domain attenuates the inhibitory effects of QKI-6 on cell proliferation and migration. The cells were overexpressed the wild-type or mutant of QKI-6. ns, no significance, **P < 0.01, ***P < 0.001 and ****P < 0.0001 with *t*-test. (I and J) Significant interaction effect between QKI-6 and PABPN1 LLPS on cell proliferation (I, *n* = 6) and migration (J, *n* = 5). Cells were transfected with CMV-Myc or Myc-QKI-6 and Flag-PABPN1-WT or Flag-PABPN1-AP. **P < 0.01 and ****P < 0.0001 with two-way ANOVA test.

To explore the role of QKI-6 in APA, we employed a dual-luciferase reporter system incorporating proximal poly(A) sites derived from the WAPAL and CTNNBIP1 genes (Hu et al., 2022). The usage rate of proximal poly(A) sites was measured by the hRluc/hluc ratio (Fig. 4A). Co-transfection of the bicistronic reporter and QKI-6 into HEK293T cells can significantly reduce the hRluc/hluc ratio compared to NC overexpression (*P < *0.001 for CTNNBIP1 and *P < *0.01 for WAPAL with *t*-test) (Fig. 4B), suggesting that QKI-6 inhibits the usage of proximal poly(A) sites. To determine whether the repressive effect of QKI-6 on the proximal poly(A) sites usage is mediated by PABPN1, we co-transfected QKI-6 and the bicistronic reporter containing the CTNNBIP1 proximal poly(A) site into HEK293T cells with or without PABPN1 knockdown. In PABPN1-knockdown cells, QKI-6 overexpression fails to reduce the hRluc/hluc ratio, indicating a loss of its repressive effects on proximal poly(A) site usage (Fig. 4C). Analysis of variance (ANOVA) analysis further reveals a significant interaction effect (*P < *0.01) between QKI-6 and PABPN1 on APA regulation.

It has been found that QKI knockdown induces global 3′ UTR shortening in human mammary epithelial cells as revealed by 3′ end-anchored RNA sequencing (Neumann et al., 2024). Using this data, we identified an enrichment of genes linked to colorectal cancer and the Wnt signaling pathway among those exhibiting APA switching (Fig. S14). We then selected four genes (NRAS, PML, BIRC5, and RAB8B) to further validate the regulatory role of QKI-6 and PABPN1 on APA using qRT-PCR in HEK293T cells (Fig. 4D). For each gene, two pairs of primers were designed to amplify the common and extended regions of the 3′ UTR. In cells without PABPN1 knockdown, QKI-6 overexpression can increase the extended/common ratios for all genes, indicating that the 3′ UTRs are lengthened. However, PABPN1 knockdown attenuates the effect of QKI-6 on APA regulation. ANOVA analysis reveals a significant interaction effect between QKI-6 and PABPN1 on the APA of all four genes (Fig. 4D). These findings from the bicistronic reporter system and endogenous genes provide direct evidence that QKI-6 regulates APA through its interaction with PABPN1.

Next, we investigated the role of QKI/PABPN1 axis in colorectal cancer cell proliferation and migration. Knockdown of QKI in colorectal cancer cell lines SW480 and SW620 can significantly increase cell proliferation (*P <* 0.001) and migration rates (*P <* 0.01 or *P <* 0.001), as measured by the CCK-8 and Transwell assays, respectively (Fig. 4E and 4F). Conversely, overexpression of QKI-6 in SW480 and SW620 significantly inhibits both cell proliferation (*P < *0.0001 or *P < *0.01) and migration (*P < *0.001 or *P <* 0.0001) (Fig. 4G and 4H). Notably, overexpression of QKI-6-ΔKH and QKI-6-G118R mutants fails to exert significant effects on cell proliferation and migration (Fig. 4G and 4H), suggesting that the KH domain of QKI-6 is crucial for its inhibitory effects on colorectal cancer cell growth and migration.

We further examined whether the inhibitory effects of QKI-6 on cancer cell proliferation and migration depend on PABPN1 LLPS. SW480 and SW620 cells were co-transfected with QKI-6 and either PABPN1-WT or the LLPS-deficient mutant of PABPN1-AP. In cells overexpressing PABPN1-AP, the inhibitory effects of QKI-6 on proliferation and migration are significantly reduced compared to those in cell with overexpression of PABPN1-WT (*P < *0.01 or *P < *0.0001 with ANOVA) (Fig. 4I and 4J). These results indicate that the inhibitory effects of QKI-6 on colorectal cancer cell proliferation and migration are also dependent on PABPN1 LLPS.

### Reduced nuclear localization of QKI attenuates PABPN1 LLPS

**Figure 5.**
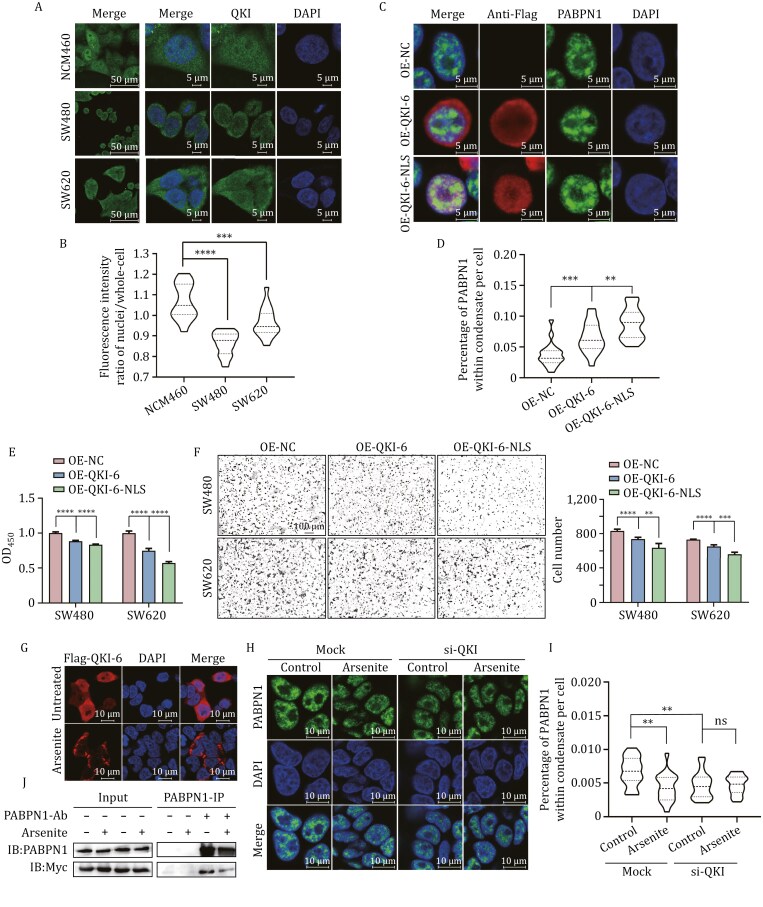
**The nuclear localization of QKI affects PABPN1 LLPS.** (A) Representative IF images show different subcellular distribution of endogenous QKI in NCM460, SW480 and SW620 cells. (B) The ratio of nuclear to whole-cell distribution of endogenous in NCM460, SW480 and SW620 cells. The fluorescence intensity of QKI was quantified using the ImageJ software. *n* = 18. ***P < 0.001 and ****P < 0.0001 with *t*-test. (C) Representative IF images of SW480 cells with overexpressed pCMV-Flag, Flag-QKI-6 and Flag-QKI-6-NLS. Cells were stained with antibodies against PABPN1 and Flag. (D) The percentage of PABPN1 within the condensate in SW480 cells with overexpressed pCMV-Flag, Flag-QKI-6 and Flag-QKI-6-NLS. *n* = 20. **P < 0.01 and ***P < 0.001 with *t*-test. (E and F) The effects of Flag-QKI-6 and Flag-QKI-6-NLS overexpression on cell proliferation and migration measured by CCK-8 (*n* = 6, E) and Transwell assays (*n* = 5, F). **P < 0.01, ***P < 0.001 and ****P < 0.0001 with *t*-test. (G) The effect of arsenite treatment on localization of Flag-QKI-6 in SW480 cells. SW480 cells were transfected with Flag-QKI-6 and treated with 500 μmol/L NaAsO_2_ for 30 min after 24 h of transfection. Cells were stained with anti-Flag to visualize Flag-QKI-6 localization. (H) Representative IF images show the effects of QKI and arsenite on PABPN1 LLPS in SW480 cells. After 48 h of transfection with si-NC or si-QKI, the cells were treated with 500 μmol/L NaAsO_2_ for 30 min. PABPN1 was visualized by staining with anti-PABPN1. (I) The percentage of PABPN1 within the condensate of cells with knockdown of QKI and arsenite treatment. *n* = 18. **P < 0.01, ns, not significant with *t*-test. (J) Co-IP analysis reveals that arsenite treatment can disrupt the interaction between PABPN1 and QKI-6. HEK293T cells were transfected with Myc-QKI-6 for 24 h, followed by treatment with 500 μmol/L NaAsO_2_ for 30 min. Co-IP was performed using an anti-PABPN1 antibody, with a homologous IgG antibody serving as a control.

In addition to the reduced expression of QKI in colorectal cancer cells, we observed differences in its subcellular distribution between colorectal cancer cells (SW480 and SW620) and normal cells (NCM460). IF imaging analysis reveals a significantly lower nuclear-to-whole-cell ratio of endogenous QKI in SW480 and SW620 cells compared to NCM460 cells (*P <* 0.001 or *P < *0.0001) (Fig. 5A and 5B). Given the reduced LLPS droplets of PABPN1 in colorectal cancer cells (Hu et al., 2024), we further investigated whether enhanced nuclear localization of QKI promotes PABPN1 LLPS. To this end, we overexpressed Flag-tagged QKI-6-WT and QKI-6-NLS in SW480 and SW620 cells, respectively. IF analysis with anti-Flag shows that the addition of an NLS sequence significantly enhances nuclear localization of QKI-6 (Figs. 5C and S12A). Further IF analysis with anti-PABPN1 reveals that cells overexpressing QKI-6-NLS exhibit a significantly higher percentage of PABPN1 in LLPS droplets compared to those overexpressing QKI-6-WT (*P <* 0.01) (Figs. 5D and S12B). Additionally, QKI-6-NLS overexpression significantly reduces proliferation (*P < *0.0001) and migration rates (*P < *0.01 or *P < *0.001) in SW480 and SW620 cells compared to overexpression of QKI-6-WT (Fig. 5E and 5F).

Previous studies have reported that QKI-6 and G3BP1 co-localize within cytosolic stress granules under arsenite-induced stress (Zhao et al., 2023). To explore this further, we transfected Flag-QKI-6 into SW480 cells and treated them with 500 μmol/L NaAsO_2_ for 30 min. As expected, Flag-QKI-6 localizes to both the nucleus and cytoplasm under normal conditions but predominantly relocated to the cytoplasm following arsenite treatment (Fig. 5G), indicating arsenite-induced nuclear-cytoplasmic shuttling of QKI-6. We examined the effect of arsenite treatment on PABPN1 LLPS in SW480 cells with or without QKI knockdown. IF analysis with anti-PABPN1 shows a significant reduction in PABPN1 LLPS upon arsenite treatment (*P < *0.01) (Fig. 5H and 5I). However, this attenuation effect is abolished in QKI-knockdown cells (Fig. 5H and 5I). Additionally, Co-IP analysis with anti-PABPN1 reveals that the amount of QKI-6 pulled down by PABPN1 is markedly decreased in arsenite-treated cells compared to the control (Fig. 5J). Collectively, these findings indicate that arsenite-induced cytoplasmic localization of QKI-6 represses PABPN1 LLPS.

## Discussion

Here, we developed a high-throughput approach by combining CRISPR/Cas9, BiFC, FACS and second-generation sequencing to screen protein regulators of LLPS. With this new method, we screened the sgRNA library targeting RBPs for the regulators of PABPN1 LLPS, and identified that QKI can promote PABPN1 LLPS by enhancing its RNA-binding affinity. Furthermore, the reduced expression level and nuclear localization of QKI in colorectal cancer cells negatively impacts PABPN1 LLPS, subsequently leading to increased usage of proximal APA sites and enhancing the proliferation and migration rates of cancer cells.

Many proteins have been found to undergo LLPS, and the alteration of LLPS is involved in various biological processes (Mitrea et al., 2022). Protein LLPS can be regulated by post-translational modifications, other proteins, RNAs, and environmental factors such as ionic strength, pH, and molecular crowding (Adame-Arana et al., 2020; Nishi et al., 2010). In this context, identifying the regulators of protein LLPS is therefore crucial for elucidating its regulatory mechanisms and developing tools to modulate LLPS. Recently, several groups have used high-content imaging to screen small molecules that modulate protein LLPS (Lim et al., 2023; Wang et al., 2023; Wheeler et al., 2020; Xie et al., 2022). For example, Wheller et al. developed Craft-ID by combining high-content imaging, CRISPR/Cas9, and microraft array techniques to screen RBPs involved in stress granule formation (Wheeler et al., 2020). Colonies on microrafts were screened using high-content imaging, and positive colonies with granule alterations were isolated and sequenced one by one—an approach that is both time- and cost-intensive. BiFC has emerged as a powerful tool to study protein-protein interactions (Kerppola, 2008). Lim et al. demonstrated that BiFC with the Venus fluorescence protein can monitor the oligomerization state of Tau protein and screened the small molecules affecting Tau protein oligomerization and aggregation using BiFC combined with high-content imaging (Lim et al., 2023). Here, we fused split fragments of Venus with PABPN1 and validated that the BiFC can monitor PABPN1 LLPS (Fig. 1B–H). By knocking down the known regulator SNRPD2, we found a robust correlation among PABPN1 LLPS droplets, the fluorescence intensity of PABPN1-BiFC, and SNRPD2 expression levels (Fig. 1I–L). Combining BiFC with CRISPR/Cas9, FACS, and high-throughput sequencing, we developed the new method to efficiently screen for proteins regulating the LLPS of target proteins.

PABPN1, an inhibitor of proximal poly(A) site usage, plays an important role in many physiological and pathological processes by regulating APA. We observed a reduction in the LLPS of PABPN1 but not in its expression level in cancer cells and identified SNRPD2 and SNRNP70 as regulators of its phase separation (Hu et al., 2022, 2024). Additionally, PABPN1 LLPS has been shown to affect mouse oocyte development (Dai et al., 2022), and alanine-expanded PABPN1 forms toxic aggregates causing OPMD (Banerjee et al., 2013). In this study, we screened an sgRNA library targeting 1,484 RBP genes for regulators of PABPN1 LLPS and found that PAPD5, ZCCHC14, HNRNPL and QKI promote PABPN1 LLPS, while PAPD7 and MRPL53 repress it (Fig. 2E–G). Further studies are needed to determine the mechanism underlying the regulation of PABPN1 by these proteins. These findings expand our understanding of the regulatory landscape governing PABPN1 LLPS and may provide insight into the roles of PABPN1 LLPS in cancer and OPMD.

QKI, a known tumor suppressor, is downregulated in colorectal cancer, and its promoter methylation is a potential biomarker for early disease detection (Yang et al., 2010; Zhang et al., 2022). We identified that QKI directly interacts with PABPN1 using Co-IP assays (Fig. 3B–D). This interaction is mediated by the KH domain of QKI (Fig. 3E and 3F) and the EP domain and C-terminal of PABPN1 (Fig. 3I and 3J), as shown using domain deletion mutants. A recurrent mutation G118R in the KH, identified through the cBioPortal database (Fig. 3G), disrupts this interaction (Fig. 3H). Consistent with the importance of the KH domain for QKI-PABPN1 interaction, KH domain deletion and the G118R mutation fail to promote PABPN1 LLPS (Figs. 3L, 3M and S12). These results demonstrate that QKI promotes PABPN1 LLPS through its interaction with PABPN1 mediated by the KH domain. We further found that QKI can enhance the RNA binding affinity of PABPN1 (Fig. S13A–C), which may promote PABPN1 LLPS.

While this study establishes QKI as a critical regulator of PABPN1 LLPS, several factors should be considered further. Arsenite treatment or QKI overexpression, could introduce other variables that may affect the observed LLPS changes of PABPN1. Additionally, the combined effect of QKI, SNRPD2, SNRNP70 and other proteins should be investigated in future studies.

It has been found that QKI knockdown can lead to global 3′ UTR shortening in human mammary epithelial cells (Neumann et al., 2024). We also validated the effect of QKI on repressing the usage of proximal poly(A) sites with a bicistronic reporter and endogenous genes (Fig. 4B–D). Overexpressing QKI in cells with or without PABPN1 knockdown revealed that the regulation of QKI on APA is mediated by PABPN1 (Fig. 4C and 4D). Knockdown, overexpression and the KH domain mutant of QKI showed that QKI represses cell proliferation and migration in colorectal cancer cells (Fig. 4E–H), and these effects are also PABPN1-dependent to a certain extent (Fig. 4I and 4J).

In addition to the observed reduction in QKI expression, we also found a decrease in the nuclear localization of QKI in colorectal cancer cells (Fig. 5A and 5B). Interestingly, QKI’s nuclear translocation has been associated with diverse cellular processes, including apoptosis regulation and viral infection responses (Pilotte et al., 2001; Sánchez-Quiles et al., 2011). The reduced nuclear localization of QKI likely diminishes its ability to promote PABPN1 LLPS in cancer cells, as validated by overexpression of QKI and QKI-NLS (Figs. 5C, 5D and S12), and further supported by the effect of arsenite treatment (Fig. 5H and 5I).

QKI has previously been shown to regulate tumorigenesis and progression through regulating alternative splicing (de Miguel et al., 2016; Novikov et al., 2011). The reduced expression of QKI in epithelial-mesenchymal transition was found to promote the proximal APA sites (Neumann et al., 2024). In this study, we found that the reduced QKI expression and nuclear localization in colorectal cancer cells disrupt PABPN1 LLPS, promoting the usage of proximal poly(A) sites, cell proliferation and migration. This provides a novel mechanism underlying QKI’s tumor-suppressive effects in cancer cells.

In conclusion, we developed a high-throughput method for screening protein regulators of target protein LLPS and successfully identified that QKI can promote PABPN1 LLPS. Furthermore, the reduction of QKI expression and nuclear localization in colorectal cancer cells leads to decreased PABPN1 LLPS and shortened 3′ UTRs of other genes, which further promotes cell proliferation and migration. Given the prevalence and importance of LLPS in gene transcription, cell signal transduction and kinds of diseases such as cancer, neurodegenerative diseases and inflammation, it is necessary to elucidate their regulation mechanism. Our new screening method offers an efficient tool for such investigations and for rapidly identifying new targets.

## Materials and methods

### Cell culture

HEK293T cells were cultured in Dulbecco’s Modified Eagle Medium (DMEM, Gibco) supplemented with 10% fetal bovine serum (FBS) (FSP500, ExCell Bio) at 37°C in a 5% CO_2_ humidified incubator. SW480 and SW620 cells were cultured in RPMI Medium 1640 (Gibco) supplemented with 10% FBS. The culture medium was refreshed every 2 days, and the cells were subcultured every three days. The passage rate for HEK293T cells ranged from 8% to 10%, while for SW480 and SW620 cells, it ranged from 15% to 20%.

### Plasmid construction

Truncated Venus (VN173, VC155) was fused to the N- or C-terminal of PABPN1, and cloned into pCDH-CMV-MCS using the CloneUFO^TM^ One Step Cloning Kit (C101, ATG Biotechnology, China). Domain deletion mutants of QKIs (ΔC, ΔC&QUA1, ΔC&KH, ΔC&QUA2, ΔC&PY-rich) were cloned into pCMV-(DYKDDDDK)-N, and domain deletion mutants of PABPN1s (ΔEP, ΔCCD, ΔRRM, ΔC) were cloned into pCMV-Myc. *E*. *coli* DH5α Competent cells (AG11806, ACCURATE BIOTECHNOLOGY (HUNAN) CO., LTD, ChangSha, China) and LB medium were used for transformation. The primer sequence was provided in Table S2.

### Bimolecular fluorescence complementation (BiFC)

We overexpressed 1 μg Myc-tagged PABPN1-VN173/VN173-PABPN1 and Flag-tagged PABPN1-VC155/VC155-PABPN1 in HEK293T cells. After 24 h of transfection, the cells were fixed with 4% paraformaldehyde (Solarbio), stained with DAPI (C0065, Solarbio) to label nuclei, and imaged using confocal microscopy. For BiFC signal detection, the excitation wavelength was set to 515 nm, and fluorescence emission was collected using the HyD detector in the range of 528 nm to 572 nm. The confocal pinhole size was set to 151.6 µm, corresponding to 999.97 mAU (Airy units), which allowed for optimal signal detection in our imaging system. Multiple fields of view were acquired to confirm the reproducibility of the observed fluorescence patterns.

For the generation of a stable PABPN1-BiFC cell line, we cloned the constructs into the pCDH-CMV-MCS vector and packaged them into lentiviral particles using a three-plasmid system. The supernatant containing the lentivirus was collected and used to infect SW480 cells that stably express Cas9. After 48 h of infection, fluorescence-positive cells were sorted using flow cytometry. This sorting process was repeated twice to enrich the desired population. Single-cell clones were then isolated, and the expression levels of PABPN1-VN173 and PABPN1-VC155, as well as the phase separation of PABPN1-BiFC, were validated using Western blot and immunofluorescence. Clones with comparable expression levels of PABPN1-VN173 and PABPN1-VC155 and phase separation states closely resembling the endogenous condition were selected for subsequent experiments. For subsequent IF experiments, cells were treated with 1,6-hexanediol or sorted by flow cytometry, immediately fixed with 4% paraformaldehyde, and stained with DAPI for imaging.

### siRNA and plasmid transfection

SW480 cells were seeded on the plate 24 h prior to transfection, ensuring a cell confluency of 30%–40%. Transfection was performed using RNAiPro Transfection Reagent (MIKX Co., Ltd, MK4018) and Opti-MEM^®^ Medium, following the protocol, with siRNA at a final concentration of 50 nmol/L. The siRNA target sequences, provided in Table S3, were synthesized by Beijing Tsingke Biotech Co., Ltd. The culture medium was refreshed after 6 h, and the follow-up experiment was conducted 48 h later. For plasmid transfection, 1 μg of QKI plasmid and 2 μg of PABPN1 plasmid, were mixed with jetPRIME^®^ transfection reagent (Polyplus) following the protocol, respectively. The culture medium was refreshed after 4 h, and the follow-up experiment was conducted 24–48 h later.

### Recombinant protein expression and purification of EGFP-PABPN1

DNA fragments were cloned into the pET-32a-EGFP vector (digested with *Eco*RⅠ and *Xho*Ⅰ). Protein expression was carried out in *E. coli* BL21(DE3) cells, which were grown in LB medium at 37°C with shaking until the OD_600_ reached 0.6–0.8. Expression was induced by adding 0.6 mmol/L IPTG, followed by overnight incubation at 20°C with agitation at 210 rpm.

Cells were collected by centrifugation at 8,000 rpm for 6 min, and the pellet was resuspended in 50 mL of Binding Buffer. Lysis was performed using a high-pressure cell disruptor, and the lysate was clarified by centrifugation at 10,000 rpm for 20 min. The supernatant was filtered through a 0.45 µm Millipore filter and subjected to purification using a Ni-NTA metal affinity column. Finally, the purified proteins were concentrated and buffer-exchanged into a high-salt solution via ultrafiltration.

### Arsenite treatment

Cells were subjected to arsenite treatment by exposing them to a solution containing 500 μmol/L sodium arsenite for a duration of 30 min. The treatment was conducted at physiological conditions, with the cells maintained at 37°C in a humidified atmosphere containing 5% CO_2_.

### Immunofluorescence staining

SW480 and SW620 cells were pre-seeded on 15 mm glass bottom cell culture dishes (801002, NEST Biotechnology) and briefly rinsed with ice-cold PBS. Subsequently, the cells were fixed with 4% fixative solution (Salarbio, P1110) for 10 min and washed three times with PBS. To permeabilize the cells, 0.1% Triton X-100 in PBS was applied for 15 min, followed by blocking with 1% BSA, 22.52 mg/mL glycine in PBST (PBS + 0.1% Tween 20) for 30 min. For cell staining, the cells were incubated overnight with the primary antibody in PBST, followed by three washes with PBST. The antibodies utilized for staining comprised of anti-PABPN1 (rabbit, A1735, Abclonal), anti-Myc (mouse, Sigma), and anti-Flag (mouse, Sigma), anti-QKI (rabbit, Immunoway). Next, the cells were incubated with the Alexa Fluor™ 488 or 568 secondary antibodies (Thermo Fisher) in PBST for 1 h, and washed three times with PBST. Please note that the aforementioned steps are not necessary if the transfected protein fused with a fluorescent protein. To stain the cell nuclei, DAPI at a concentration of 1 μg/mL was applied for 1 min, followed by rinsing with PBS. Finally, the cells were imaged using a Leica TCS SP8 STED 3X microscope (Leica Microsystems). The statistical analysis of subcellular distribution and the percentage of protein within condensate is performed using ImageJ.

### Image analysis

Immunofluorescence images were analyzed using Fiji/ImageJ. To calculate the percentage of PABPN1 within condensates per cell, individual cells were segmented and added to the ROI Manager. The total fluorescence intensity of PABPN1 was quantified by measuring the integrated density (IntDen). Images were converted to 8-bit format, and an appropriate threshold was applied to distinguish droplets from the background. The fluorescence intensity of droplets was measured using the “Analyze Particles” module. The percentage of PABPN1 within condensates was determined by dividing the droplet fluorescence intensity by the total cell fluorescence intensity.

For the nuclear-to-whole-cell fluorescence intensity ratio, each cell and its nucleus were outlined separately and added to the ROI Manager. Fluorescence intensities were measured using the “Measure” function, and the ratio was calculated by comparing nuclear fluorescence intensity per unit area to whole-cell fluorescence intensity per unit area.

### Fluorescence recovery after photobleaching

Cells were cultured on 15 mm glass bottom cell culture dishes (801002, NEST Biotechnology). Myc-PABPN1-VN173 and Flag-PABPN1-VC155 were transfected into the cells. FRAP experiments were performed using a TCS SP8 STED 3X microscope equipped with a 100×, N.A. 1.4 oil-immersion apochromate objective. Individual cells were focused at a resolution of 256 × 256, and a circular ROI area with a diameter of 1 was designated. The detector was set as the PMT, and the fluorescence in the ROI region was bleached using a 488 nm laser at 100% laser intensity. Two images were taken before bleaching, and 30 images were taken during fluorescence recovery after bleaching.

### Flow cytometry

SW480 cells were treated with 0.05% trypsin at 37°C for 2 min, and the trypsin activity was neutralized by adding fresh RPMI 1640 complete media. Subsequently, the cells were centrifuged at 300 ×*g* for 5 min, and the supernatant solution was aspirated. The pelleted cells were resuspended in PBS, ensuring thorough mixing. To obtain a single-cell suspension, the cell suspension was then passed through Cell Strainers and filtered. For fluorescence intensity analysis of the cells, the CytoFLEX Flow Cytometer (Beckman Coulter) was employed, followed by data processing using the CytExpert software. In the case of FACS (fluorescence-activated cell sorting), the Beckman MoFlo Astrios EQs (Beckman Coulter) instrument was utilized for sorting single cells for CRISPR/Cas9 screening. Data analysis was performed using the Summit software.

Our FACS method follows a systematic gating strategy to ensure accurate cell sorting. First, cells were gated based on forward scatter (FSC) and side scatter (SSC) to isolate the main population of interest, as larger and more complex cells exhibit higher FSC and SSC values. Single cells were then identified by excluding doublets or clumps using FSC-A vs. FSC-H and SSC-A vs. SSC-W plots, where single cells align along the diagonal, while doublets appear off-diagonal. For cells expressing PABPN1-BiFC, fluorescence intensity was analyzed using the SSC-H vs. 513/26-Height-Log plot, and the top and bottom 10% of cells were selected based on fluorescence intensity. This selection captures phenotypic extremes to enrich cells with the strongest and weakest PABPN1 phase separation, improving the signal-to-noise ratio and enabling robust genotype–phenotype association analysis. Furthermore, to maintain coverage of approximately 500 cells per sgRNA in our CRISPR/Cas9 library (9,099 unique sgRNAs), we sorted about 1 × 10^7^ cells for each fraction from total 1 × 10^8^ cells. These detailed gating strategies and selection criteria ensure reproducibility and accuracy in downstream analyses.

### Western blot

2 × 10^6^ cells were lysed using 200 μL of RIPA buffer and then incubated on ice for 20 min. Once completely lysed, the cell lysate was centrifuged at 16,000 ×*g* for 15 min. The supernatant was collected, and protein quantification was performed between samples using the Pierce™ BCA Protein Assay Kits (Thermo Scientific). Cell lysate was mixed with SDS-PAGE Sample Loading Buffer (Sangon Biotech), and the sample was then boiled at 100°C for 15 min. SDS-PAGE gels were utilized for protein isolation, followed by the transfer of the proteins to a nitrocellulose filter membrane at 90 V for 90 min. Subsequently, the membrane was blocked for 1 h in 5% skim milk powder (dissolved in TBST). The primary antibodies used in western blotting were anti-Myc (mouse, 60003-2-IG, proteintech), anti-Flag (mouse, dia-an), anti-β-actin (mouse, FD0060, Fudebio, Hangzhou, China), anti-GAPDH (mouse, proteintech), and anti-PABPN1 (rabbit, A1735, Abclonal). The membrane was incubated with the primary antibodies at room temperature for 2 h or overnight at 4°C. After washing the membrane three times with 1× TBST, the membrane was incubated with the secondary antibodies at room temperature for 1 h. The secondary antibody was anti-rabbit/mouse IgG, HRP-linked Antibody (CST). Finally, the immunoreactive bands were detected using the ECL Kit (Merck Millipore) and a Chemiluminescent Imaging System (Tanon).

### Reverse transcription and quantitative real-time PCR (qRT-PCR)

Total RNA was extracted using the phenol-chloroform method. 2 × 10^6^ cells were washed with 1× PBS and then lysed with 1 mL TRIzol (Thermo Fisher) for 5 min. To extract phenol, 200 μL trichloromethane was added, followed by the precipitation of RNA using 400 μL isopropyl alcohol. After washing the RNA with 75% ethyl alcohol, the RNA precipitates were dissolved in DEPC water. 400 ng RNA was reverse transcribed using the TransScript^®^ Uni All-in-One First-Strand cDNA Synthesis SuperMix for qPCR (One-Step gDNA Removal) (AU341-02, TransGen Biotech, China). Quantitative real-time PCR was performed using SYBR Green Premix Pro Taq HS qPCR Kit (AG11701, Accurate Biotechnology (Hunan) Co., Ltd., ChangSha, China) in a 384-well plate and LightCycler^®^ 480 II (Roche) under the following conditions: pre-incubation at 95°C for 30 s for 1 cycle, amplification at 95°C for 5 s and then at 60°C for 40 s for 40 cycles, melting curve at 95°C for 5 s, at 60°C for 60 s and continuously from 60°C to 90°C with 1 cycle, and cooling at 50°C for 30 s with 1 cycle. Finally, the data was analyzed using the 2^–ΔΔCt^ method. The primer sequence was provided in Table S4.

### RNA immunoprecipitation qPCR (RIP-qPCR)

Cells were crosslinked with 0.1% formaldehyde at room temperature (RT) for 10 min. Crosslinking was quenched by adding 125 mmol/L glycine and incubating at RT for 5 min. Cells were then pelleted by centrifugation at 500 ×*g* for 5 min, washed twice with cold 1× PBS, and centrifuged again to remove the supernatant. The cell pellets were lysed on ice for 10 min using RIPA lysis buffer. After lysis, samples were centrifuged to collect the supernatant. A portion of the supernatant was reserved as input, while the remaining was incubated with Dynabeads Protein G (Sigma) pre-bound with the specific antibody (incubated at RT for 1 h). The mixture was incubated at 4°C for 2 h with gentle rotation.

The beads were then washed sequentially with RIPA lysis buffer and high-salt RIPA buffer to remove nonspecific binding. For crosslink reversal, reverse-crosslinking buffer was added to the beads, and the samples were incubated at 55°C for 1 h. RNA was extracted from the samples using Trizol reagent. The enrichment of target RNA was assessed by qRT-PCR using specific primers, and the relative enrichment efficiency of the target gene was calculated.

### Co-immunoprecipitation (Co-IP)

2 × 10^6^ cells were lysed by adding 400 μL of IP lysis buffer at 4°C for 20 min. At the same time, Protein G Magnetic Beads (MCE, HY-K0202) were incubated with the antibody at room temperature for 1 h with rotation. The antibodies used in Co-IP included anti-PABPN1 (Abclonal), anti-Myc (Sigma), anti-Flag (Sigma), and anti-IgG (CST). After centrifugation, 40 μL of the supernatant was mixed with 10 μL of SDS-PAGE Sample Loading Buffer (Sangon Biotech) and boiled at 100°C for 15 min to obtain the input. Then, 300 μL of the supernatant from the cell lysate was added to the beads-antibody mixture and incubated with rotation for 2 h. Following 3 washes with washing buffer for 5 min each, the beads were resuspended in 50 μL of 1× loading buffer and boiled at 100°C for 15 min. The detection of protein bands was carried out using Western blot.

### Lentivirus production and CRISPR/Cas9 screening

Lentivirus was produced in HEK293T cells by transfecting plasmids using the jetPRIME^®^ transfection reagent (Polyplus). The plasmids used were PSPAX2, PMD2.G, and PCDH. Lentiviral supernatant was collected 48–72 h after transfection and filtered through a 0.45 μm filter. Virus concentrator was added and thoroughly mixed with the supernatant. The lentivirus solution was then incubated at 4°C for 24 h before being centrifuged at 1,500 ×*g* for 50 min. To resuspend the lentiviruses, 1 mL of RPMI 1640 media was used. The solution was stored at − 80°C for later use.

For sgRNA library lentivirus production, 12 μg of lenti-sgRNA-puro was transfected into 10 cm plates of cells. To estimate the MOI (multiplicity of infection), lentivirus packed with 0, 0.1, 1, 5, 20, and 50 μL was added to infect cells for 24 h, respectively. Puromycin at a concentration of 1 μg/mL and 1% penicillin/streptomycin (Gibco) were added to select positive cells. The lentiviral titer was determined by the concentration at which there was no cell death observed. sgRNA lentiviral libraries at MOI = 0.2–0.3 (500 times the library complexity) were used to infect SW480 cells in 30 cm dishes. After 7 days of puromycin selection, FACS (fluorescence-activated cell sorting) was performed.

A PCR-based library preparation method was used to amplify DNA fragments coding the sgRNAs, employing a single-index library construction approach (Fig. S5). Primers were designed with complementary sequences targeting the U6 promoter and gRNA scaffold regions in the lenti-gRNA-puro vector. These primers also incorporated P5 and P7 adapter sequences to enable library binding and cluster generation on the sequencing flow cell. Additionally, sequencing primer binding sites (Rd1 SP and Rd2 SP) and the index for sample differentiation were positioned between the P7 adapter and Rd2 SP, ensuring compatibility with downstream sequencing workflows.

### Cell proliferation and migration assay

For the cell proliferation analysis, cells were seeded at a density of 8,000 cells per well in a 96-well plate. The cells were cultured in 100 μL of RPMI Medium 1640 supplemented with 10% FBS. Subsequently, the cells were incubated at 37°C with 5% CO_2_ for 24 h. After that, 10 μL of Enhanced Cell Counting Kit-8 (C0041, Beyotime) was added to each well of the medium. Following a 2-h incubation period, the optical density at 450 nm (OD_450_) was measured. Each treatment was performed in six replicates.

For the cell migration analysis, a total of 2 × 10^4^ cells were seeded in the upper chambers of Transwell inserts with a pore size of 8 μm (Corning). The upper chambers contained 200 μL of RPMI Medium 1640 without FBS, while the bottom chambers were filled with 600 μL of RPMI Medium 1640 supplemented with 20% FBS. The cells were then cultured at 37°C with 5% CO_2_ for 1–16 h. After the incubation, the cells were fixed with 4% fixative solution (Salarbio, P1110) for 15 min and stained with 0.1% crystal violet for 15 min. Cell counting was performed in five randomly selected fields.

### Statistical analysis

For comparisons between two groups, a two-tailed Student’s *t*-test was used to determine statistical significance. For experiments involving two independent variables, a two-way ANOVA was conducted to assess the main effects of each factor as well as their interaction effects, where the interaction term evaluates whether the effect of one factor depends on the level of the other factor. Data are expressed as the Mean ± SD. These analyses were conducted using GraphPad Prism software (GraphPad).

For CRISPR screening data, sequencing reads were analyzed using the MAGeCK algorithm to identify differentially selected genes. Briefly, read counts from each sample were median-normalized to account for variations in library sizes and read count distributions. The variance of read counts was estimated by borrowing information across sgRNAs, and a negative binomial (NB) model was employed to test for significant differences in sgRNA abundance between the two cell populations. sgRNAs were ranked based on P-values calculated from the NB model, and genes with consistently higher or lower sgRNA rankings than expected were identified using α-RRA method. Correlation coefficient of sgRNA reads number between two replicates were calculated. We then tested the correlation difference between sgRNAs with and without significant changes using a permutation test. We resampled the sgRNAs without replacement for 1000 times, and for each time we calculated the difference of correlation coefficients of the first 1,248 sgRNAs and the other sgRNAs using R software. The percent of iterations with greater absolute values of difference than the observed value was calculated as *P* value.

## Supplementary Material

pwaf022_suppl_Supplementary_Figures_S1-S14_Tables_S2-S3

pwaf022_suppl_Supplementary_Tables_S1

## Data Availability

All the Illumina-type sequencing data are available in China National Center for Bioinformation with accession number PRJCA027345.
