# Supplementary material for: BiFC and FACS-based CRISPR screening revealed that QKI promotes PABPN1 LLPS in colorectal cancer cells
Source: Protein Cell. 2025 Mar 6;16(7):557–74. doi: 10.1093/procel/pwaf022 (PMC12275093; doi:10.1093/procel/pwaf022)
Supplement: pwaf022_suppl_Supplementary_Figures_S1-S14_Tables_S2-S3 [file pwaf022_suppl_supplementary_figures_s1-s14_tables_s2-s3.pdf]

Supplementary information

Supplement Fig 1. Venus fragments fused to the N- or C-terminus of PABPN1 affect its LLPS.
Supplement Fig 2. Dynamics of endogenous PABPN1 LLPS under 1,6-HEX treatment.
Supplement Fig 3. Establishment of SW480 cell lines stably expressing Cas9 and PABPN1-BiFC.
Supplement Fig 4. The effect of SNRPD2 on PABPN1 LLPS.
Supplement Fig 5. Schematic of a PCR-based library preparation method for sgRNA amplification.
Supplement Fig 6. Correlation analysis of sgRNA counts between cell fractions and replicates.
Supplement Fig 7. sgRNA read counts for the SNRPD2 gene in the Top and Bottom groups.
Supplement Fig 8. The interference efficiency of candidate genes measured by qRT-PCR.
Supplement Fig 9. Reduced expression level of tumor suppressor QKI in colorectal cancer.
Supplement Fig 10. The interaction between PABPN1 and three isoforms of QKI.
Supplement Fig 11. The schematic diagram depicting the glutamate to alanine mutations in the EP domain of PABPN1.
Supplement Fig 12. QKI-6 and its nuclear localization promotes PABPN1 LLPS in SW620 cells.
Supplement Fig 13. QKI-6 enhances the RNA binding affinity of PABPN1.
Supplement Fig 14. KEGG pathway enrichment analysis of genes with significant APA changes following QKI knockdown.
Supplement Table 1. The list of sgRNAs targeting 1,484 genes coding for RNA-binding proteins.
Supplement Table 2. PCR primer sequence for plasmid construction
Supplement Table 3. siRNAs
Supplement Table 4. qRT-PCR primer sequence

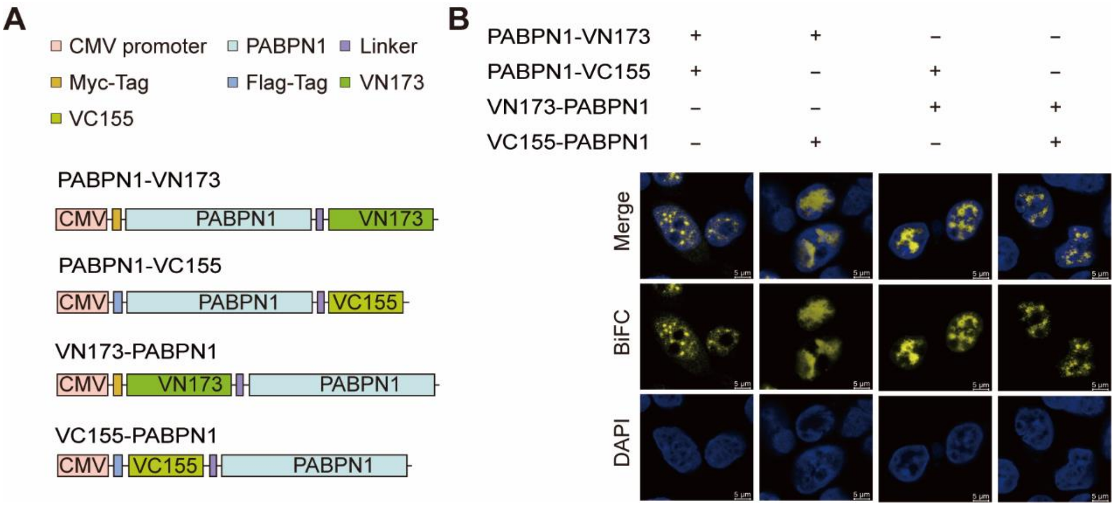

Supplement Fig 1. Venus fragments fused to the N- or C-terminus of PABPN1 affect its LLPS.

(A) Schematic diagram of different combinations of complementation constructs, showing VN173 or VC155 fused to the N- or C-terminus of PABPN1.

(B) Confocal microscopy images of HEK293T cells transfected with the complementation constructs depicted in (A). Cells were fixed 24 hours post-transfection, and nuclei were stained with DAPI.

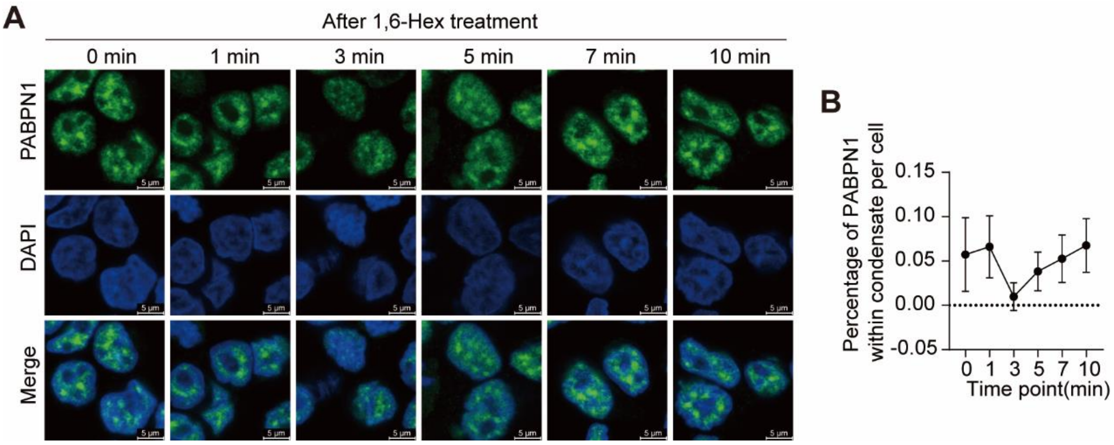

Supplement Fig 2. Dynamics of endogenous PABPN1 LLPS under 1,6-HEX treatment.

(A) Representative IF images of endogenous PABPN1 in SW480 cells with treatment of 1.5% 1,6-HEX at different time points. SW480 cells were treated with 1.5% 1,6-HEX, and then stained with anti-PABPN1 and DAPI.

(B) The percentage of PABPN1 within condensates at different time points under 1.5% 1,6-HEX treatment. n = 21.

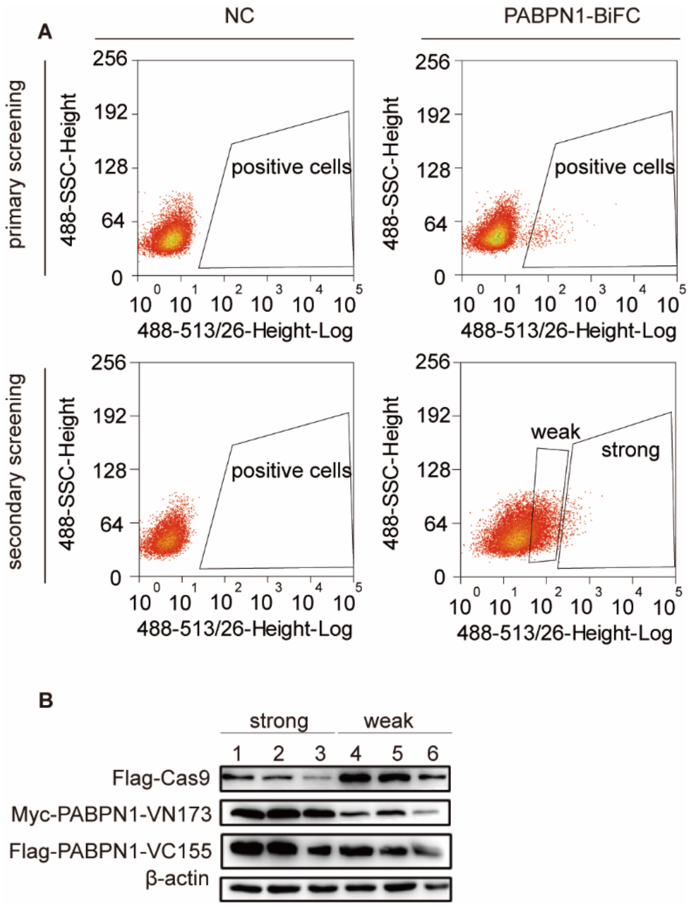

Supplement Fig 3. Establishment of SW480 cell lines stably expressing Cas9 and PABPN1-BiFC.

(A) Representative flow cytometry scatter plots used to sort monoclonal SW480 cells stably expressing PABPN1-BiFC. "Strong" indicates cells with high fluorescence intensity, while "Weak" indicates cells with low fluorescence intensity.

(B) Western blot analysis showing the expression levels of Flag-Cas9, Myc-PABPN1-VN173, and Flag-PABPN1-VC155 in monoclonal cell lines. Anti-Myc and anti-Flag antibodies were used to detect Flag-Cas9, Myc-PABPN1-VN173, and Flag-PABPN1-VC155, respectively, with β-actin probed as a loading control.

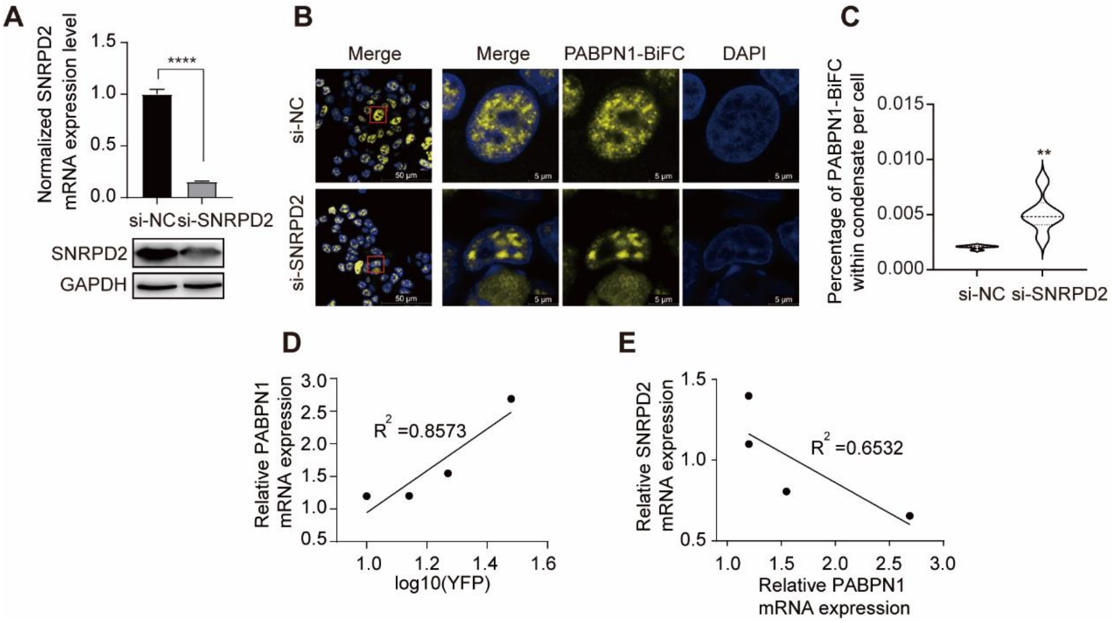

Supplement Fig 4. The effect of SNRPD2 on PABPN1 LLPS.

(A) Knockdown efficiency of SNRPD2 in SW480 cells measured by qRT-PCR. SW480 cell lines stably expressing Cas9 and PABPN1-BiFC was transfected with si-NC or si-SNRPD2. Data are presented as mean ± SD. n = 3. ****p < 0.0001 with t-test. POLR2L was used as internal inference for qRT-PCR. GAPDH was used as the internal reference for Western Blot.

(B) Representative confocal microscopy images showing PABPN1-BiFC LLPS in SW480 cells with SNRPD2 knockdown. SW480 cell lines stably expressing Cas9 and PABPN1-BiFC was transfected with si-NC or si-SNRPD2. Cells were fixed 48 hours post-transfection, and nuclei were stained with DAPI.

(C) Quantification of the percentage of PABPN1-BiFC within condensates in cells with SNRPD2 knockdown. The statistical data is derived from five large-field IF images. Data are presented as mean ± SD. **p < 0.01 with t-test.

(D) Linear regression analysis of PABPN1 expression levels and fluorescence intensity of PABPN1-BiFC. SW480 cells stably expressing PABPN1-BiFC were transfected with si-SNRPD2 and sorted into four groups using FACS. Relative expression levels of SNRPD2 were measured by qRT-PCR, with si-NC serving as the negative control.

(E) Linear regression analysis of SNRPD2 expression levels and PABPN1 expression levels. Experimental treatments were performed as described in (D).

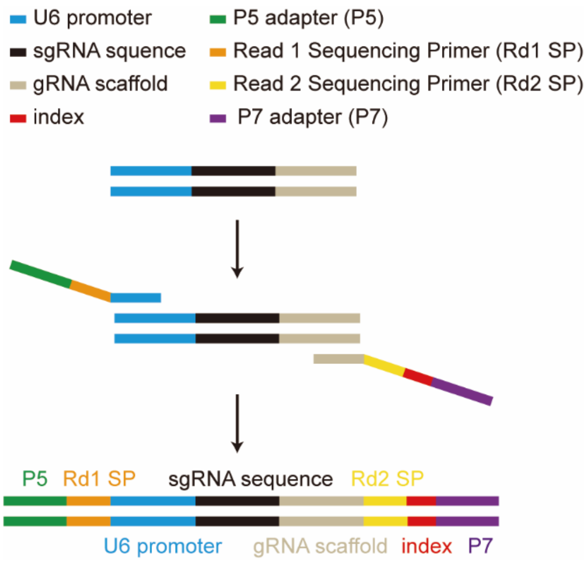

Supplement Fig 5. Schematic of a PCR-based library preparation method for sgRNA amplification. Complementary sequences were designed to target the U6 promoter and gRNA scaffold within the lenti-gRNA-puro vector. The primers were engineered to include these complementary sequences, along with P5 and P7 adapter sequences to enable library binding and cluster generation on the flow cell. Additionally, sequencing primer sites (Rd1 SP and Rd2 SP) and sample differentiation indices are positioned between the P7 adapter and Rd2 SP.

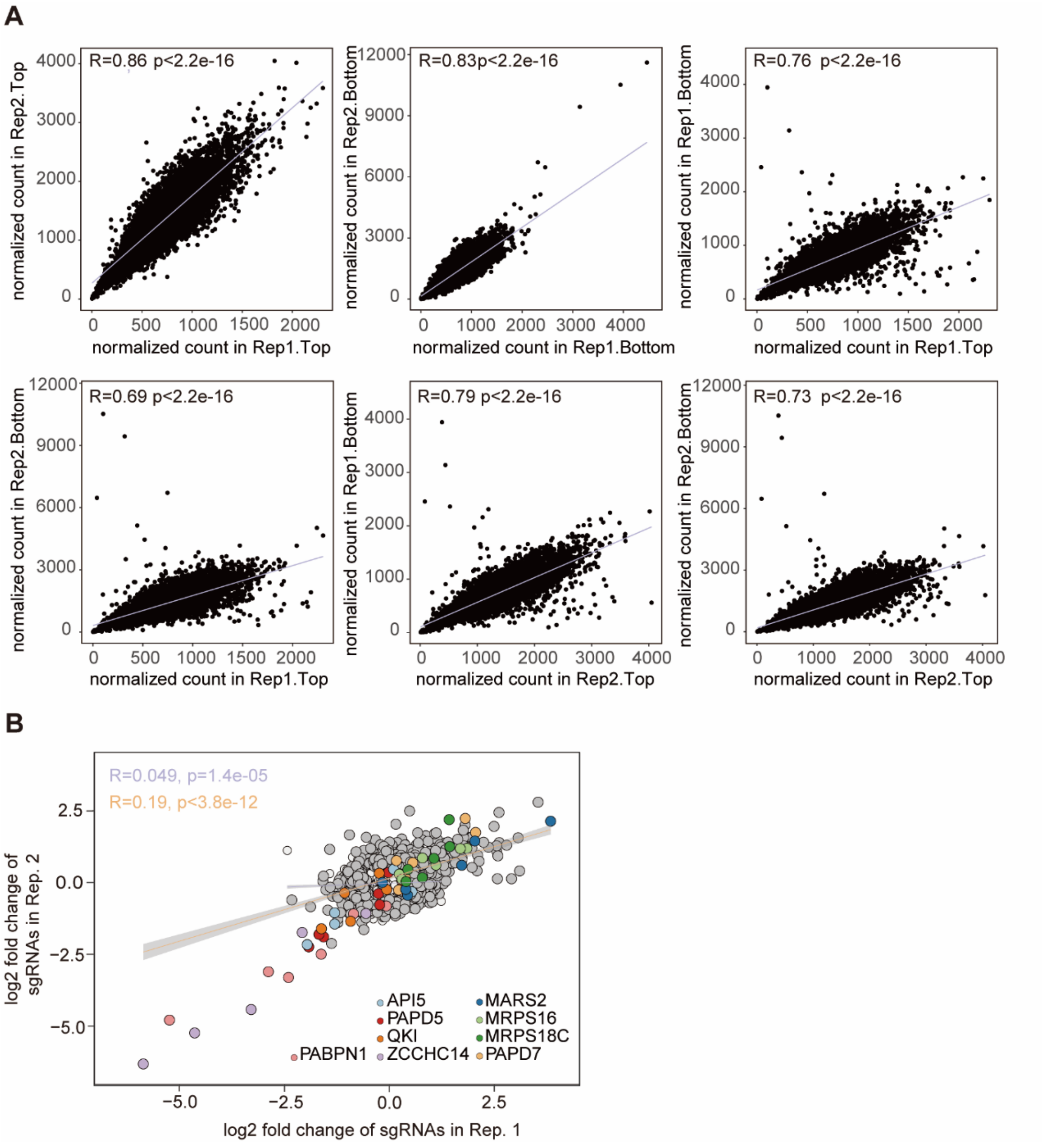

Supplement Fig 6. Correlation analysis of sgRNA counts between cell fractions and replicates.

(A) The detailed correlation analysis of normalized sgRNA counts between samples.

(B) Correlation coefficient (R) of fold changes for the Top/Bottom groups of each sgRNA between two replicates. sgRNAs with significant Top/Bottom fold changes (FDR < 0.01) are shown as gray points, with their regression line represented by the yellow line. Non-significant sgRNAs are depicted as white points, and the regression line for all sgRNAs is shown in purple.

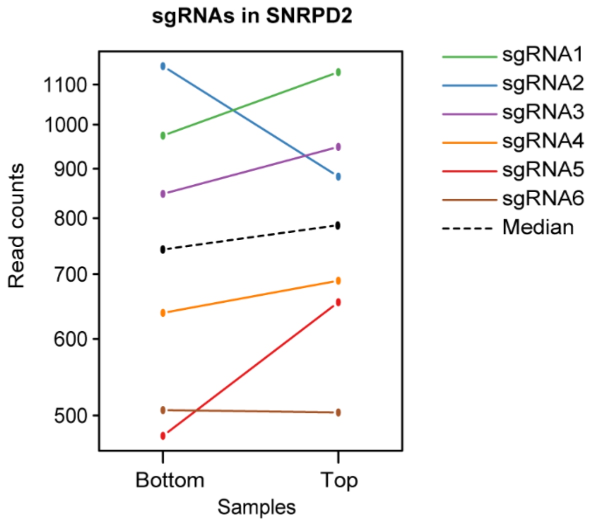

Supplement Fig 7. sgRNA read counts for the SNRPD2 gene in the Top and Bottom groups. The dashed line indicates the median value.

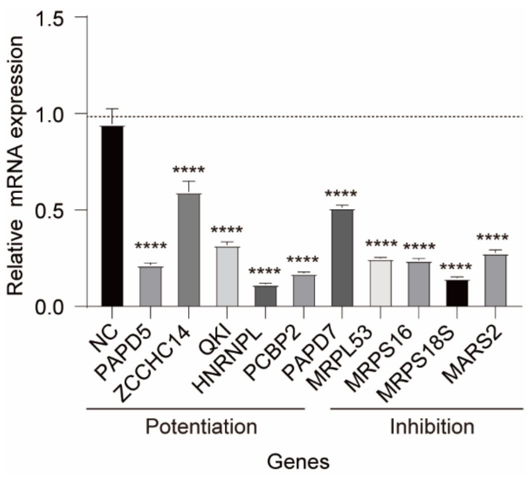

Supplement Fig 8. The interference efficiency of candidate genes measured by qRT-PCR. SW480 cells were transfected with siRNAs targeting the candidate genes, with si-NC serving as the negative control. POLR2L was used as internal inference for qRT-PCR. ****$p < 0.0001$ with t-test. Data are presented as mean ± SEM.n = 3.

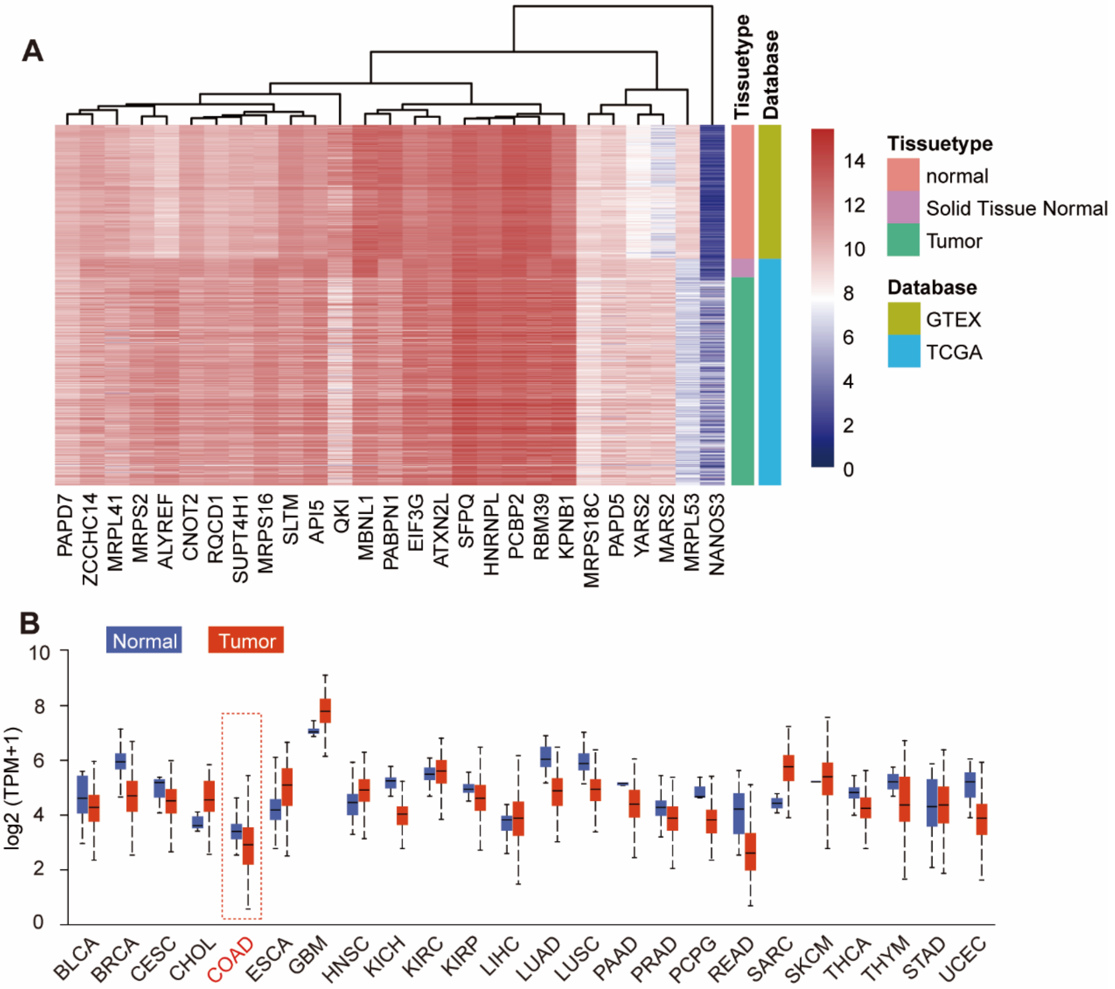

Supplement Fig 9. Reduced expression level of tumor suppressor QKI in colorectal cancer.

(A) Heatmap showing the mRNA expression levels of candidate genes in colorectal tumor and adjacent normal tissues, based on data from the GTEx and TCGA databases. **(B)** Boxplot illustrating the mRNA expression levels of QKI in tumor versus adjacent normal tissues. Expression levels are presented as log2(TPM + 1).

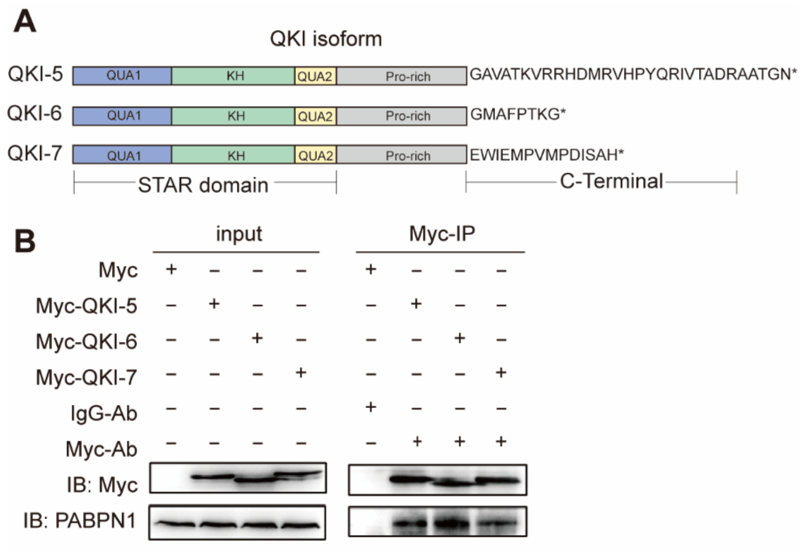

Supplement Fig 10. The interaction between PABPN1 and three isoforms of QKI.
(A) Schematic representation of the domain structures of the three QKI isoforms.
(B) Co-immunoprecipitation (Co-IP) assay demonstrating the interaction between PABPN1 and the three QKI isoforms. Myc-tagged QKI isoforms were transfected into HEK293T cells. Anti-Myc was used for immunoprecipitation (IP), and anti-PABPN1 was used to detect endogenous PABPN1.

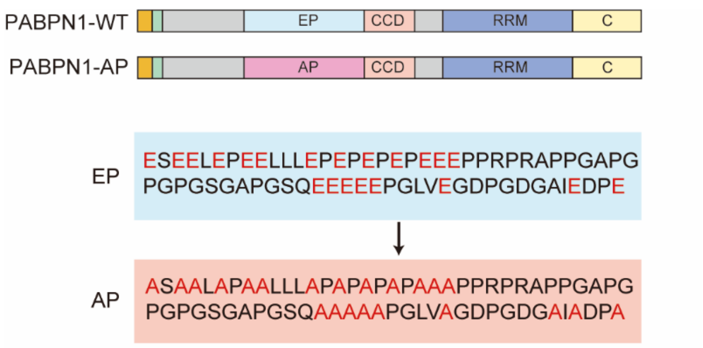

Supplement Fig 11. The schematic diagram depicting the glutamate to alanine mutations in the EP domain of PABPN1.

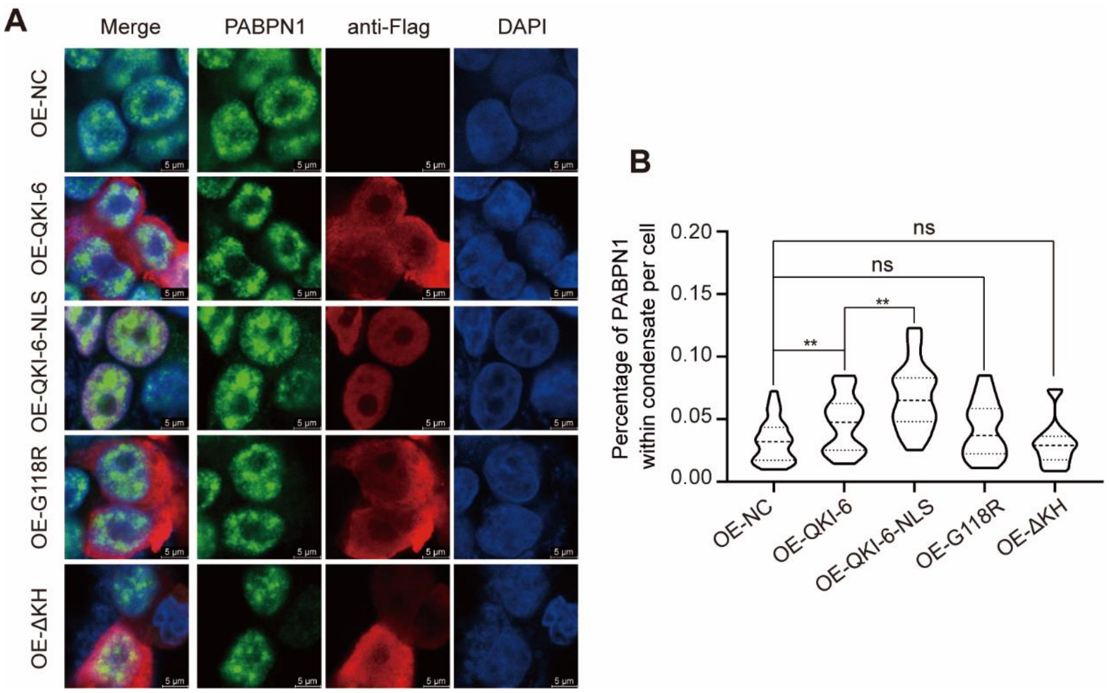

Supplement Fig 12. QKI-6 and its nuclear localization promotes PABPN1 LLPS in SW620 cells.

(A) Representative immunofluorescence (IF) images illustrating PABPN1 LLPS in SW620 cells. Cells were transfected with CMV-Flag, Flag-QKI-6, Flag-QKI-6-NLS, Flag-QKI-6-ΔKH, or Flag-QKI-6-G118R. After 24 hours, the cells were stained using anti-Flag and anti-PABPN1 antibodies.

(B) Quantification of the percentage of PABPN1 within condensate in SW620 cells overexpressing CMV-Flag, Flag-QKI-6, Flag-QKI-6-NLS, Flag-QKI-6-ΔKH, or Flag-QKI-6-G118R. n = 20. ns, no significance and $^{**}p < 0.0001$ with t-test.

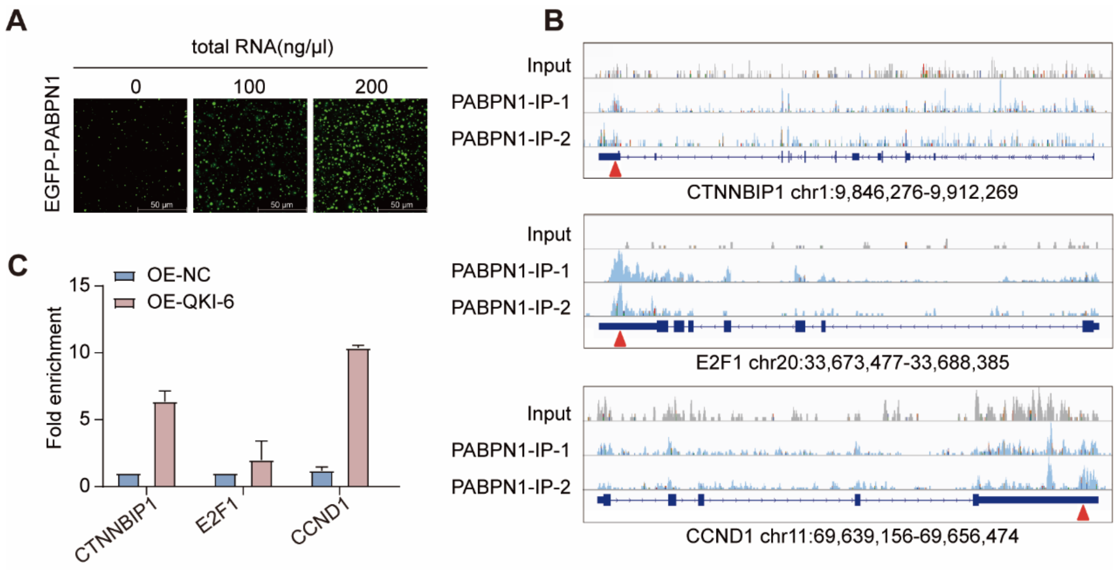

Supplement Fig 13. QKI-6 enhances the RNA binding affinity of PABPN1.

(A) Confocal microscopy images show the effect of RNA on PABPN1 LLPS. Recombinant EGFP-PABPN1 protein was expressed and purified in vitro. EGFP-PABPN1 (final concentration: 5 μM) was incubated with 10% PEG8000 and RNA at varying concentrations (0, 100, and 200 ng/μL).

(B) Integrative Genomics Viewer (IGV) visualization of PABPN1 binding sites at the CTNNBIP1, E2F1, and CCND1 genes based on eCLIP analysis. The red arrows indicate the positions of primer designs.

(C) RNA immunoprecipitation followed by quantitative PCR (RIP-qPCR) analysis of PABPN1 binding to the CTNNBIP1, E2F1 and CCND1 genes in SW480 cells transfected with CMV-Flag or Flag-QKI-6, along with Myc-PABPN1. Anti-Myc antibodies were used to pull down RNAs bound by PABPN1, while IgG served as a negative control. Fold enrichment of RNA pulled down by anti-Myc compared to IgG is shown.

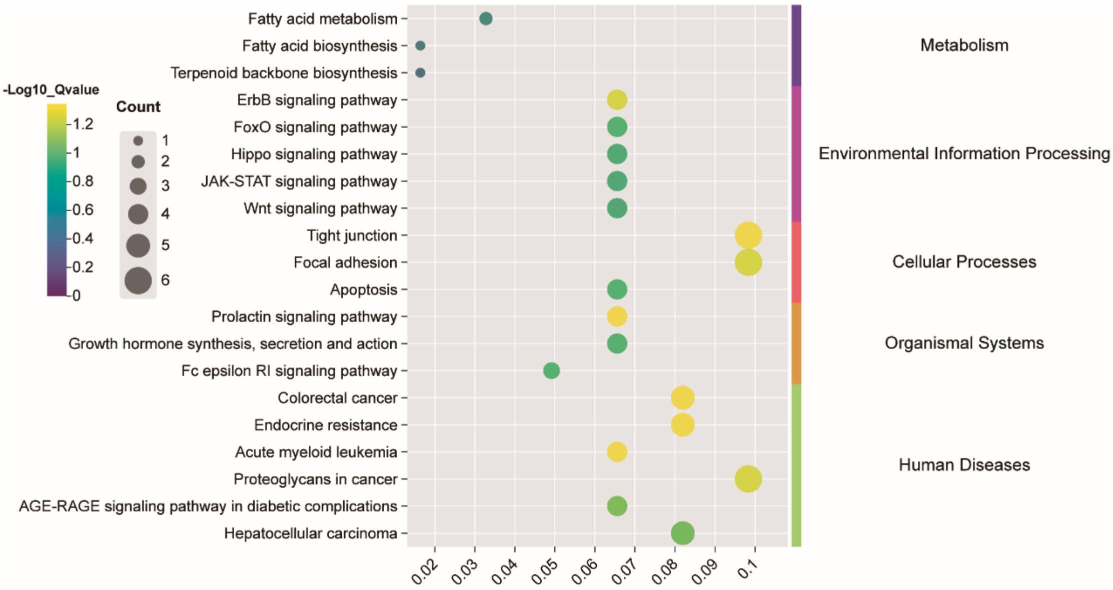

Supplement Fig 14. KEGG pathway enrichment analysis of genes with significant APA changes following QKI knockdown.

Supplement Table 2. PCR primer sequence for plasmid construction

Primer name	Sequence
Flag-PABPN1-VC155-F1	TGAACCGTCAGATCCGCTAGCATGGACTACAAAGACGATGACGACAAGGAATTCATGGC
Flag-PABPN1-VC155-F2	GACGACAAGGAATTCATGGCGGCGGCGGCGGCGG
Flag-PABPN1-VC155-F3	GTCGACCGCAGCATTGCGACCGACAAGCAGAAGAACGGCATCAAGGCCAACTTCAAGAT
Flag-PABPN1-VC155-F4	CAAGGCCAACTTCAAGATCCGCCACAACATCGAGGACGGCGGCGTGCAGCTCGCCGACC
Flag-PABPN1-VC155-R1	GGTCGCAATGCTGCGGTCGACGTAAGGGGAATACCATGATG
Flag-PABPN1-VC155-R2	TTATCTAGATCCGGTGGATCCTTACTTGTACAGCTCGTCCATGCCGAGAGTGATCCCGG
Myc-PABPN1-VN173-F1	TGAACCGTCAGATCCGCTAGCATGGCATCAATGCAGAAGCTGATCTCAGAGGAGGACCT
Myc-PABPN1-VN173-F2	CTGATCTCAGAGGAGGACCTGGAATTCATGGCGGCGGCGGCGGCGGC
Myc-PABPN1-VN173-F3	AAAATTCCGAACGATCTGAAACAGAAAGTGATGAACCATATGGTGAGCAAGGGCGAGGA
Myc-PABPN1-VN173-R1	TTCAGATCGTTCGGAATTTTGCACGCCGGGCGGTCGACGTAAGGGGAATACCATGATGT
Myc-PABPN1-VN173-R2	TTATCTAGATCCGGTGGATCCTTACTCGATGTTGTGGCGGATCTTGAAGTTGGCCTTGA
PCDH-Flag-PABPN1-VC155-F1	GACGATGACGACAAGGAATTCATGGCGGCGGCGGCGGCGGCGGCAGCA
PCDH-Flag-PABPN1-VC155-F2	ATAGAAGATTCTAGAGCTAGCATGGACTACAAAGACGATGACGACAAGGAATTCATGGC
PCDH-Flag-PABPN1-VC155-F3	CACGCAGCATTGCGACCGACAAGCAGAAGAACGGCATCAAGGCCAA
PCDH-Flag-PABPN1-VC155-R1	TTGTCGGTCGCAATGCTGCGTGCGGCCGCGTAAGGGGAATACCATGATGTCGCTCTAG
PCDH-Flag-PABPN1-VC155-R2	ATCCAGAGGTTGATTGTCGACTTACTTGTACAGCTCGTCCATGCCGAGAGTGATCCCGG
PCDH-Myc-PABPN1-VN173-F1	AATGCAGAAGCTGATCTCAGAGGAGGACCTGGAATTCATGGCGGCGGCGGCGGCGGCGG
PCDH-Myc-PABPN1-VN173-F2	ATAGAAGATTCTAGAGCTAGCATGGCATCAATGCAGAAGCTGATCTCAGA
PCDH-Myc-PABPN1-VN173-F3	GCACGCCCGGCGTGCAAAATTCCGAACGATCTGAAACAGAAAGTGATGAACCATATGGT
PCDH-Myc-PABPN1-VN173-R1	GAATTTTGCACGCCGGGCGTGCGGCCGCGTAAGGGGAATACCATGATGTCGCTCTAGCC
PCDH-Myc-PABPN1-	ATCCAGAGGTTGATTGTCGACTTACTCGATGTT

VN173-R2	GTGGCGGATCTTGAAGTTGGCCTTGA
Myc-QKI-F	TGGCCATGGAGGCCCGAATTCACATGGTCGGG GAAATGGAAACGAAGGAG
Myc-QKI-5-R	CCGCGGCCGCGGTACCTCGAGTTAGTTGCCGG TGGCGGCTCG
Myc-QKI-6-R	CCGCGGCCGCGGTACCTCGAGTTAGCCTTTCG TTGGGAAAGCCATACC
Myc-QKI-7-R	CCGCGGCCGCGGTACCTCGAGTCAATGGGCTG AAATATCAGGCATGA
Flag-DsRed-QKI-6-ΔC-F1	ACGACAAGCTTATGCGAATTCACATGGCCTCC TCCGAGAACGTCATCAC
Flag-DsRed-QKI-6-ΔC-F2	CAAAATTCCGAACGATCTGAAACAGAAAGTG ATGAACCATATGGTCGGGGAAATGGAAA
Flag-DsRed-QKI-6-ΔC-R1	TCAGATCGTTCGGAATTTTGCACGCCGGGCGT GAACCACCGCCTCCGCCCAGGAACAGG
Flag-DsRed-QKI-6-ΔC-R2	CCGCGGCCGCGGTACCTCGAGTTATAATACAC CACTAGGTTCAATAGGATAC
Flag-DsRed-QKI-6-ΔC&QUA1-F	AAAATTCCGAACGATCTGAAACAGAAAGTGAT GAACCATTTACAAGAGAAACTTTATGT
Flag-DsRed-QKI-6-ΔC&KH-F	GCAGCAGAAGGAGAAGACAGCCT
Flag-DsRed-QKI-6-ΔC&KH-R	AGGCTGTCTTCTCCTTCTGCTGCCTGAACAAT AGGTCCCACAGCAT
Flag-DsRed-QKI-6-ΔC&QUA2-F	GATGCCAACATTAAATCACCAGCCC
Flag-DsRed-QKI-6-ΔC&QUA2-R	GGGCTGGTGATTTAATGTTGGCATCAGGTACC AATAATTTCTTCACTTCTTC
Flag-DsRed-QKI-6-ΔC&PY-R	CCGCGGCCGCGGTACCTCGAGTTATCTGTAGG TGCCATTCAGAATCGCAAGCTCCATCA
Myc-QKI-6-G118-F	GTAAAATCATGGTCCGAGGCAAA
Myc-QKI-6-G118-R	TTTGCCTCGGACCATGATTTTACATCTGGTTTC TGCTTCAAGTTGTTTGG
Flag-QKI6-NLS-R1	ACACCTTCCTCTTTTTCTTAGGGCTTCCACCTC CTCCGCCTTTCGTTGGGAAAGCCATA
Flag-QKI6-NLS-R2	CCGCGGCCGCGGTACCTCGAGTTACACCTTCC TCTTTTTCTT

Supplement Table 3. siRNAs.

Name	Target sequence
siRNA-PAPD5	GGTGAAAGTTTTAGACAAA
siRNA-HNRNPL	CATCATGCCTGGTCAGTCA
siRNA-QKI	CTGATGCTGTGGGACCTATTG
siRNA-PCBP2	GCATTAGCCTGGCTCAATA
siRNA-MARS2	GGATTCGTTTCCTGTATCT
siRNA-MRPS16	CACCTCTCTAAGCCTATGGAA
siRNA-MRPS18C	GCATTTATGGAAGGCACATTA
siRNA-MRPL53	TCATCTCACCGCTCTGGAAAT
siRNA-ZCCHC14-1	GTCAGTAATAGTTTGGAGAAT
siRNA-ZCCHC14-2	GGAGCGAAACCACGTGGATCT
siRNA-ZCCHC14-3	GGAAACTGCGTTTGCACAAGT
siRNA-PAPD7-1	CCGGTATTAGAATCAAAGA
siRNA-PAPD7-2	CGGATCGAAACTGTGGTGAAA
siRNA-PAPD7-3	GGAGGTGGATCAAAGAGAAGT
siRNA-PABPN1	CATACTGTGTGACAAATTT

Supplement Table 4. qRT-PCR primer sequence

Primer name	Sequence
POLR2L-qF	TCACTTGTGGCAAGATCGTCG
POLR2L-qR	GGGTGCATAATTGAGCAGCTTC
SNRPD2-qF	AGTCAAGAACAATACCCAAGTGC
SNRPD2-qR	ATGTTGCAGTGCCTATCGAAG
PABPN1-qF	CATGGCTGTGGTTCAGTCAACC
PABPN1-qR	CTAAGGCCAAGGAAGTCCTCAC
ZCCHC14-qF	ACCCCGTCTTTAAGCAGCTC
ZCCHC14-qR	GGCACCGTCTCTCTGACTTC
PAPD5-qF	GTCGCAGATGAGGATTCGGT
PADP5-qR	TGAGGTCAGCTGCTCTCACG
HNRNPL-qF	GCGTGAACAGTGTGCTTCTC
HNRNPL-qR	CCCCATTGAGAGAGGCCTTG
QKI-qF	TACAGACCGCTGTCATGCC
QKI-qR	AGCTGGTGCCAATGTGTAGG
PCBP2-qF	CACCATCCGGCTACTTATGC
PCBP2-qR	TGGCATTAGTGGGTCCAGCC
MARS2-qF	GCACAGATTTCATCCGCACC
MARS2-qR	AGGAAACGAATCCCCCGATG
MRPS16-qF	ACTCGTTGCCCTCAACCTAG
MRPS16-qR	GCAGTCTCTCAGCATTTGTGATC
MRPS18C-qF	TGAGGACCTGCCCATTTCAA
MRPS18C-qR	AGACCTGTAATGTGCCTTCCATA
MRPL53-qF	AACGTGGAATCGACGAGGAC
MRPL53-qR	ATAATCAGGCGATGCCCGTC
PAPD7-qF	CCCACCACTTCCAGAACACT
PAPD7-qR	GGAAGACATGTGGTGGACGG
BIRC5-S-QF	GGGCCCCTTAGCAATGTCTT
BIRC5-S-QR	CACCTCTGGTGCCACTTTCA
BIRC5-L-QF	TTCTGCCACATCTGAGTCGG
BIRC5-L-QR	GGCTGGAGTGCATTTTCTGC
NRAS-S-QF	CTCACTTGGCTGTCTGACCA
NRAS-S-QR	ATGAAAAACCTGGGGTGGCA
NRAS-L-QF	GCCTGGCCAGCTGTCATAAA
NRAS-L-QR	GATCCGGCATGGTAGCCTTC
PML-S-QF	GGTGACCAGCTTGGAGTCTCTG
PML-S-QR	CCAAATGACAACTGAGGGCTGG
PML-L-QF	AGCAGTGTGGAACAGCAGAG
PML-L-QR	AGCCCATTTTACAGATGGGGAC
RAB8B-S-QF	GAGAGACTGCAGCACACCT
RAB8B-S-QR	GTGCTCTCAGCAGCAGCC
RAB8B-L-QF	AGGGACCTAGACGGACAGGA
RAB8B-L-QR	CAGAGTGAACATGACAGCCATT
